# Antecedent chromatin organization determines cGAS recruitment to ruptured micronuclei

**DOI:** 10.1038/s41467-023-36195-8

**Published:** 2023-02-02

**Authors:** Kate M. MacDonald, Shirony Nicholson-Puthenveedu, Maha M. Tageldein, Sarika Khasnis, Cheryl H. Arrowsmith, Shane M. Harding

**Affiliations:** 1 Department of Medical Biophysics, University of Toronto, Toronto, ON Canada; 2 Princess Margaret Cancer Center, University Health Network, Toronto, ON Canada; 3 Structural Genomics Consortium, University of Toronto, Toronto, ON Canada; 4 Departments of Radiation Oncology and Immunology, University of Toronto, Toronto, ON Canada

**Keywords:** Stress signalling, DNA damage response, Tumour immunology, Epigenetics

## Abstract

Micronuclei (MN) are cytosolic bodies that sequester acentric fragments or mis-segregated chromosomes from the primary nucleus. Spontaneous rupture of the MN envelope allows recognition by the viral receptor cyclic GMP-AMP synthase (cGAS), initiating interferon signaling downstream of DNA damage. Here, we demonstrate that MN rupture is permissive but not sufficient for cGAS localization. Chromatin characteristics such as histone 3, lysine 79 dimethylation (H3K79me2) are present in the nucleus before DNA damage, retained in ruptured MN, and regulate cGAS recruitment. cGAS is further responsive to dynamic intra-MN processes occurring prior to rupture, including transcription. MN chromatin tethering via the nucleosome acidic patch is necessary for cGAS-dependent interferon signaling. Our data suggest that both damage-antecedent nuclear chromatin status and MN-contained chromatin organizational changes dictate cGAS recruitment and the magnitude of the cGAS-driven interferon cascade. Our work defines MN as integrative signaling hubs for the cellular response to genotoxic stress.

## Abstract (web summary)

DNA damage-induced micronuclei are linked to downstream viral signalling through the cGAS pattern recognition receptor. Here, the authors identify features of micronuclei chromatin that determine cGAS-MN recruitment and associated pathway activation.

## Introduction

Micronuclei (MN) are acentric fragments of the nuclear genome, or whole lagging chromosomes, that were excluded from the nucleus at the end of mitosis^1,2^. These pieces of unincorporated DNA recruit an envelope that is discontinuous with the primary nuclear membrane and prone to spontaneous and irreversible loss of integrity in interphase, termed “rupture”^3–5^. MN are active participants in a DNA damage-dependent inflammatory program that has been found to accelerate tumor development, and influence both genotoxic and immune-modulatory treatment responses, in vivo^6–8^. The inflammatory program is initiated when ruptured MN are recognized by a viral pattern receptor, called cyclic GMP-AMP synthase (cGAS)^7–9^. Canonically, cGAS binds invading double-stranded (ds)DNA viruses and produces 2’3’-cyclic GMP-AMP (cGAMP) that subsequently activates stimulator of interferon genes (STING). Active STING induces type I interferon, and drives the expression of downstream interferon-stimulated genes (ISG)^10,11^. Multiple groups have demonstrated that mitotic progression is necessary for both MN formation and cGAS-dependent DNA damage-induced interferon responses^7,8^. cGAS-STING activity that is induced by exogenous DNA damage, including cancer therapeutics such as ionizing radiation (IR), drives induction of systemic immunity when genotoxic treatment is combined with immune checkpoint blockade^8,12–18^.

Despite these important consequences of cGAS recruitment to MN, the underlying features of MN that dictate cGAS recruitment and downstream interferon activity remain poorly understood. Not all MN recruit cGAS upon rupture, and the size of the cGAS-positive fraction depends on the MN-generating experimental conditions^7,19^. These observations raise the possibility that certain stress contexts are more conducive to cGAS-bound, MN-dependent inflammation than others. Several recent studies using cryogenic electron microscopy identified key cGAS protein residues that influence its binding to in vitro or nuclear chromatin, and its ability to produce cGAMP once bound^20–24^. Whether these dependencies are similarly influential when cGAS is recruited to a damaged, MN-sequestered chromatin fragment is not known. Here, we address how discrete forms of MN-generating genotoxic stress impact MN capacity to recruit and activate cGAS. We demonstrate that while MN rupture is a prerequisite for cGAS recruitment, it is not sufficient. We find that the likelihood of cGAS binding to MN upon rupture depends on the nature of the MN-inducing genotoxic stressor. We show that chromatin characteristics of the MN-sequestered DNA fragment, including histone modifications and active transcription, influence cGAS recruitment to MN and downstream inflammatory signaling. MN chromatin organization was informed primarily by the status of the nuclear genome prior to genotoxic stress. Our results identify micronuclear chromatin content as a structural link between basal nuclear characteristics and DNA damage-induced cytokine signaling.

## Results and discussion

### cGAS recognition of ruptured micronuclei depends on the inciting genotoxic stressor

**Fig. 1:**
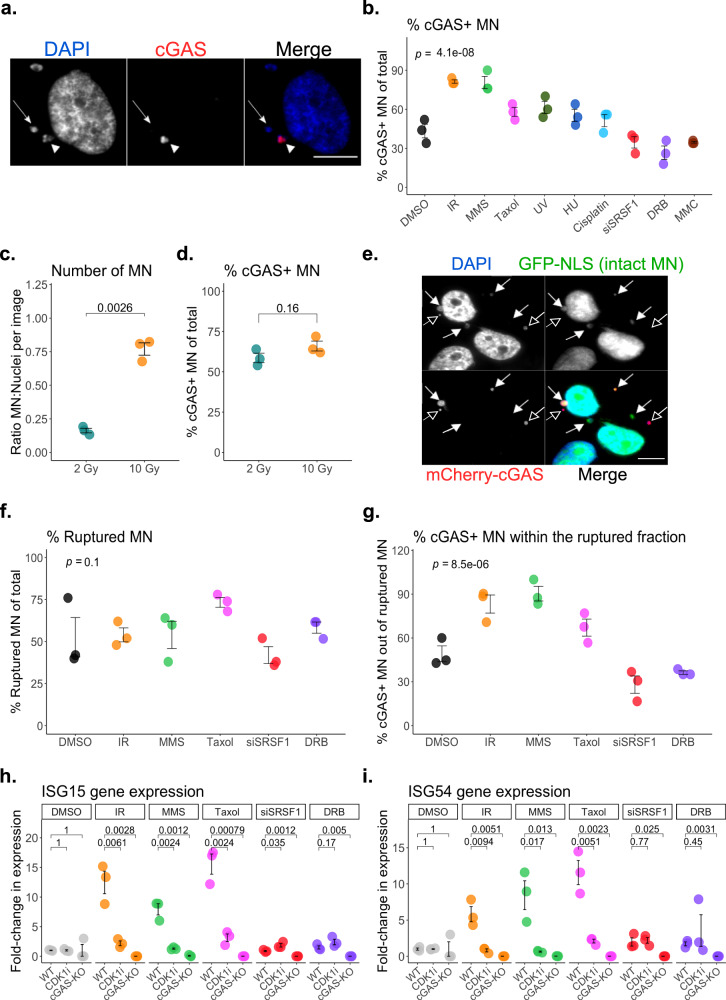
cGAS recognition of ruptured micronuclei depends on the inciting genotoxic stressor. **a** Representative cGAS+ (arrowhead), and cGAS- MN (arrow) in MCF10A. Scale bar = 10 μm. Image representative of 3 experiments. **b** Percent cGAS+ MN by immunofluorescence (IF), 72 h post-exposure in MCF10A. See Methods for details on each exposure. Statistical comparison by one-way ANOVA. **c** Ratio of MN to nuclei present in a microscopy field of view (FOV). Each point represents the mean of at least 5 FOVs, for a total of three independent biological replicates. **d** Percent cGAS+ MN by IF, 72 h post-exposure to 2 or 10 Gy ionizing radiation (IR) in MCF10A. **e** Representative intact (GFP-nuclear localization signal (NLS)+, filled arrow) and ruptured (empty arrow) MN. Scale bar = 20 μm, in MCF10A. Images representative of 3 experiments. **f** Percent ruptured (GFP-NLS-negative) MN by IF, 72 h post-exposure in MCF10A. Statistical comparison by one-way ANOVA. **g** Percentage of MN that recruit mCherry-cGAS following their rupture, measured by live-cell imaging in the mitosis following MN formation. Statistical comparison by one-way ANOVA, in MCF10A. **h**, **i** Gene expression of *ISG54* and *ISG15* by RT-qPCR, 72 h post-exposure, in wild-type (WT) or cGAS-knockout (KO) MCF10A cells. CDK1 inhibitor is used to block all MN formation by preventing cell cycle progression. See Methods for details of each exposure. All statistical comparisons performed using a two-sided Student’s *t*-test unless otherwise indicated. NS: *p* = 1, ns: *p* > 0.05, *p ≤ 0.05, **p ≤ 0.01, ***p ≤ 0.001, ****p ≤ 0.0001. All individual data points presented for immunofluorescent scoring of MN represent the mean percentage of MN that were positive for the indicated marker, from each biological replicate out of 50 total MN per replicate. All error bars represent standard error of the mean, for three independent biological replicates. Source data are provided as a Source Data file.

We and others have observed that MN can form spontaneously, form in response to several genotoxins that deposit diverse DNA lesions throughout the cell cycle, or form as a consequence of aberrant chromosomal segregation during mitosis^7–9,19,25–27^. To explore how each of these stimuli might generate MN with differential cGAS status, we developed a panel of MN-generating treatments and scored cGAS-positive MN by immunofluorescence microscopy (IF) (Fig. 1a, b and Supplementary Fig. S1a, b, c) (see Methods for a detailed description of each stressor and associated controls). In MCF10A (a diploid, immortalized, non-transformed human epithelial cell line), and in HeLa and U2OS cells (cell lines derived from human cancers), the percentage of MN that have recruited cGAS by 72 h following stress exposure demonstrates a significant association with the inciting genotoxin (Fig. 1b and Supplementary Fig. S1c). These treatments did not affect cGAS mRNA or protein levels (Supplementary Fig. S1d, e) or the time that a cell spends in interphase (Supplementary Fig. S1f). We considered that differences in MN burden across treatments might influence the capacity to recruit cGAS, but MN induced by either 2 or 10 Gy of ionizing radiation (IR) were equally likely to recruit cGAS (Fig. 1c, d), and differences in the percentage of cGAS+ MN across all agents did not correlate with their total MN burden (Supplementary Fig. S1g). These data suggested that intrinsic differences in MN induced by discrete forms of genotoxic stress impact their capacity for cGAS recruitment.

MN rupture is a known prerequisite for cGAS recruitment^7,9^. We considered the possibility that divergent cGAS recruitment to MN across stressors was reflecting differential propensities for MN rupture. We measured MN rupture by the absence of a GFP-tagged nuclear localization signal (NLS), previously established as a reliable rupture indicator (Fig. 1e)^3,7,9^. For these experiments, we relied on MCF10A cells, which harbor very little nuclear cGAS during interphase and therefore focus our analysis on cGAS recruitment following MN rupture (Supplementary Fig. S1h). To further exclude the minor fraction of unruptured MN that retain cGAS at mitotic exit (Supplementary Fig. S1i), we used live cell imaging and restricted our analysis to those MN that lose the GFP-NLS signal in interphase before recruiting mCherry-tagged cGAS^7^. Over the 72 h following stress exposure from our panel, we found no association between the MN-inducing genotoxin and either rupture frequency or time-to-rupture (Fig. 1f and Supplementary Fig. S1j). When we restricted our analysis to only interphase-rupturing MN, we continued to measure a significant association between cGAS-MN recruitment and inciting genotoxic stress condition (Fig. 1g). Thus, MN rupture is necessary but not wholly sufficient to explain the observed divergence in cGAS localization. Our results imply differences in the content of unruptured MN across cellular stress conditions that influence cGAS recruitment post-rupture.

We next asked whether the differential capacity to recruit cGAS to MN influenced cGAS-STING pathway activation, in diploid non-transformed MCF10A cells that retain cGAS-STING signaling without confounding DNA damage response defects or constitutive cytokine expression. In this system, the cellular ISG response depended on both mitotic progression to produce MN (prevented with a CDK1 inhibitor) and on cGAS expression by the cell (Fig. 1h, i)^7,8^. cGAMP production profiles, more specific to cGAS activity and upstream of ISG production, mirrored measured *ISG* expression levels (Supplementary Fig. S1k). Together, these data support cGAS+ MN as one biomarker of DNA damage-induced ISG signaling, among others^7,8,28–30^.

### Erasing H3K79me2 prior to ionizing radiation exposure reduces cGAS recruitment to micronuclei

**Fig. 2:**
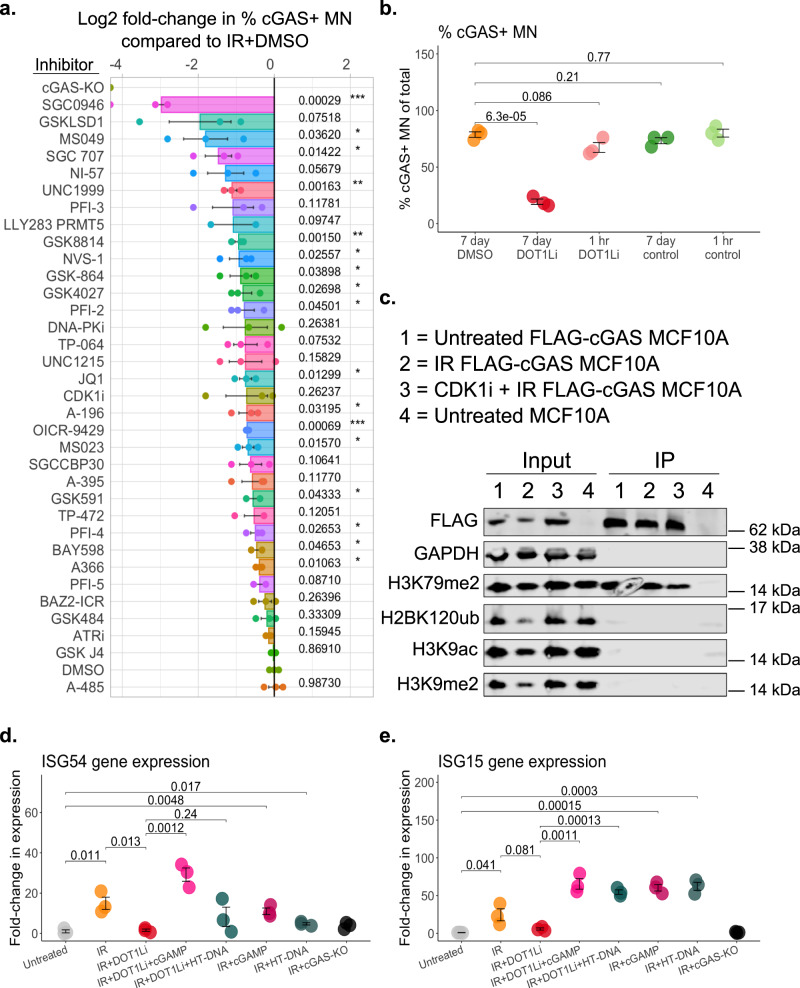
Erasing H3K79me2 prior to ionizing radiation exposure reduces cGAS recruitment to micronuclei. **a** Percent cGAS+ MN by immunofluorescence (IF), 72 h post-exposure to 10 Gy IR in MCF10A. Statistical comparisons by Student’s *t*-test using DMSO treatment as the reference group. Bonferroni-adjusted *p* values are displayed for each comparison, with statistically significant comparisons indicated with asterisks. See Supplementary Table S1 for a list of protein targets corresponding to each inhibitor in this screen. **b** Percent cGAS+ MN by immunofluorescence (IF), 72 h post-exposure to 10 Gy IR in MCF10A. Cells were pre-treated as indicated. **c** FLAG-cGAS co-immunoprecipitated (IP) from each of the indicated conditions, 72 h following 10 Gy IR. Blot representative of 3 experiments. **d**, **e** *ISG54* and *ISG15* expression by RT-qPCR, 72 h post-10 Gy IR in MCF10A. Indicated cells were pre-treated for 7 days with DOT1Li; 24 h with herring testis (HT)-DNA; or 24 h with cGAMP prior to IR exposure. All statistical comparisons performed using a two-sided Student’s *t*-test. ns: *p* > 0.05, *p ≤ 0.05, **p ≤ 0.01, ***p ≤ 0.001, ****p ≤ 0.0001. All individual data points presented for immunofluorescent scoring of MN represent the mean percentage of MN that were positive for the indicated marker, from each biological replicate out of 50 total MN per replicate. All error bars represent standard error of the mean, for three independent biological replicates. Source data are provided as a Source Data file.

Recent in vitro work has demonstrated that cGAS exhibits a binding preference for nucleosome-associated chromatin over naked dsDNA^20–24^. The chromatin features that dictate this binding preference in vivo remain unknown, particularly within MN. We asked whether the histone modifications on MN-sequestered chromatin could be influencing cGAS recruitment following rupture. We evaluated cGAS recruitment to IR-induced MN from MCF10A cells pre-treated with a library of 48 chemical inhibitors, each designed to target a specific histone reader, writer, or eraser (Supplementary Table S1)^31,32^. Five days of pre-treatment with the inhibitor library was used to fully deplete any affected chromatin marks in the nucleus, well in advance of IR exposure, ensuring that all MN-sequestered DNA fragments originated from a similarly altered nuclear chromatin pool (Supplementary Table S1, see Minimum time for reduction). We selected IR for this screen as it is known to produce a wide range of different DNA damage types randomly within the genome^33^, and because it generates a large number of MN among the highest propensity to recruit cGAS upon rupture (Fig. 1g). Several inhibitors in the screen significantly reduced cGAS-MN association after IR (Fig. 2a), an effect that was not associated with changes in MN burden (Supplementary Fig. S2a). We did not observe increased cGAS localization with any compound following IR, suggesting that cGAS recruitment is maximized under these conditions. Performing the same screen where there is minimum baseline cGAS recruitment (generating MN with 5,6-dichloro-1-beta-ribo-furanosyl benzimidazole (DRB); Fig. 1g), we observed the converse outcome of significant increases following multiple compounds but no decreases in cGAS localization (Supplementary Fig. S2b). A subset of these inhibitors was also able to increase cGAS recruitment to spontaneous MN, where cGAS recognition also tends to be low (Supplementary Fig. S2c). These results demonstrate that cGAS recruitment to MN can be manipulated with inhibitors of several histone-modifying enzymes, in a manner dependent on the inciting stress condition.

To reach and maintain a steady-state level of chromatin alteration, our experimental setup used 5 days of inhibitor treatment prior to IR exposure (Supplementary Table S1). These treatments were maintained while MN formed over 72 h post-IR, at which time we scored cGAS+ MN by IF (Fig. 2a). Therefore, there were several possible sources for the observed reductions in cGAS-MN localization. These included changes to nuclear chromatin organization, occurring prior to IR exposure; nuclear chromatin alterations caused by IR exposure; changes to damage-induced chromatin fragments that occur after their MN-sequestration; or changes to the cGAS protein itself mediated by non-histone targets of the inhibited enzyme. To delineate between these models, we interrogated the relationship between cGAS recruitment to IR-induced MN and one specific histone modification, histone 3 lysine 79 dimethylation (H3K79me2). The mono-, di-, and tri-methylation states of H3K79 are all added in procession by a single methyltransferase, disruptor of telomere silencing 1-like (DOT1L)^34^. H3K79 has no known demethylase, with all three H3K79 methylation states relying on nucleosome turnover for their removal from chromatin^35–37^. The dimethylation state in particular has been extensively characterized using the small molecule SGC0946, a DOT1L inhibitor (DOT1Li) that was the top hit in our IR-induced MN screen (Fig. 2a)^38^. The SGC0946-mediated erasure of H3K79me2 therefore represents a validated, direct, non-redundant system for this first examination of cGAS interactions with histone modifications.

Full in-cell reduction of H3K79me2 can take anywhere from 4 to 14 days of DOT1Li exposure, depending on the system^38^ (Supplementary Table S1); we found that 7 days of pre-treatment was required to fully erase H3K79me2 from the pre-damage pool of nuclear chromatin in both MCF10A and HeLa cells (Supplementary Fig. S2d, e). Full nuclear erasure of H3K79me2, established prior to IR exposure and MN formation, was necessary to prevent cGAS recruitment to subsequent MN; short pre-treatment (1 h) was not sufficient (Fig. 2b). This result argues against an acute need for DNA damage-induced DOT1L activity to promote cGAS-MN localization, and supports a preponderant role for pre-established H3K79me2 on the sequestered chromatin fragment^39^. Neither H3K79me2 erasure nor a reduction in cGAS+ MN was observed with a structurally similar inactive control compound, SGC0649 (Fig. 2b and Supplementary Fig. S2d, e)^38^ and two additional DOT1L inhibitors produced the same effect as the active SGC0946 compound (Supplementary Fig. S2f, g), as did a short hairpin (sh)RNA targeting DOT1L (Supplementary Fig. S2h, i). As a non-inhibitor approach, and to verify that the primary effect of DOT1Li was to its histone methylation rather than the methylation of non-histone targets, we used a small interfering (si)RNA targeting the ubiquitin ligase RNF20. RNF20 ubiquitinylates histone 2B, lysine 120 (H2BK120ub), which stabilizes DOT1L at the nucleosome to facilitate H3K79 methylation near the nucleosome acidic patch^40,41^. Importantly, this interaction is only known to occur at the nucleosome, not at non-histone proteins that may be targeted by DOT1L. siRNF20 caused H3K79me2 erasure and significantly reduced cGAS recruitment to IR-induced MN (Supplementary Fig. S2j, k). DOT1Li did not affect the incidence of MN rupture (Supplementary Fig. S2l) or cGAS transcript and protein levels (Supplementary Fig. S2m, n). DOT1Li-driven reduction of cGAS+ MN was also observed in irradiated HeLa and U2OS cells (Supplementary Fig. S2o), in irradiated MCF10A cells expressing a transgenic, constitutive mCherry-cGAS construct (Supplementary Fig. S2p), and in MN generated from non-IR exposures, arguing against an IR-specific effect on cGAS in the presence of DOT1Li (Supplementary Fig. S2q). FLAG-cGAS+ MN co-localized with H3K79me2 in ~50% of IR-induced MN, and both are significantly reduced in MN that are irradiated following DOT1Li (Supplementary Fig. S2r, s). FLAG-cGAS co-immunoprecipitated with H3K79me2, suggesting a possible direct binding interaction (Fig. 2c). Functionally, DOT1Li significantly impaired the IR-induced cGAS-dependent ISG response (Fig. 2d, e). This does not reflect reliance on H3K79me2 for expression of the downstream ISG pathway components, since *cGAS* and *STING* transcript levels remain intact after DOT1Li treatment (Supplementary Fig. S2m, t) and the addition of exogenous cGAMP or herring testis (HT)-DNA can elicit cGAS-STING-dependent *ISG54* and *ISG15* expression in IR+DOT1Li conditions (Fig. 2d, e)^8^. Together, these results indicate that H3K79me2-modified chromatin is a positive regulator of cGAS recruitment to IR-induced MN. Our observations are most consistent with a model where changes to nuclear H3K79me2 levels, established prior to IR exposure and MN-enclosure of chromatin fragments, are the primary contributor to reduced cGAS-MN recruitment under long-term DOT1Li conditions (Fig. 2b and Supplementary Fig. S2d–n). While our results also suggest that multiple histone targets are relevant for cGAS-MN interactions (Fig. 2a and Supplementary Fig. S2b, c), DOT1L-deposited H3K79me2 represents one determinant of cGAS-MN chromatin binding.

### Micronuclei retain chromatin features of the primary nucleus

Our observations with long-term DOT1Li suggested to us that MN carry fragments of chromatin that reflect conditions in the primary nucleus established prior to DNA damage (Fig. 2b and Supplementary Fig. S2d–k). To directly test whether local nuclear chromatin organization at the onset of DNA damage directs capacity for MN-cGAS recruitment, we used a cell-based reporter system in which DNA breaks can be induced at a defined locus upstream of a doxycycline (DOX)-inducible transcriptional unit. Upon DOX treatment, the locus transitions from a heterochromatic to a transcriptionally active state^42–44^ (Supplementary Fig. S3a). Using this system, MN are generated from the same genotoxic stress at the same genomic locus, but in the presence of distinctive nuclear organizational states (active or silenced transcription). When DOX-induced transcription is active at the time of DNA damage, the targeted-locus-containing MN were half as likely to recruit cGAS compared to MN generated when the region was transcriptionally silent (Supplementary Fig. S3b–e). Thus, the chromatin status of the MN-destined nuclear locus, established prior to DNA damage and MN-sequestration, can influence cGAS-MN interactions.

Given the observed sensitivity of cGAS to nuclear chromatin organization (Fig. 2b and Supplementary Figs. S2d–k and S3a–e) and the stress-dependent cGAS recruitment that has been previously reported (Fig. 1b, g)^19^, we asked whether specific genotoxic stress conditions were biasing the histone modifications that become MN-sequestered, influencing cGAS recruitment and ISG production (Fig. 1b, h, i). To test this possibility, we purified MN and nuclei from HeLa cells treated with five genotoxins that produce MN along the range of observed capacity for cGAS retention and profiled their histone modification profiles by mass spectrometry (Fig. 1b and Supplementary Fig. S3f). We found that the MN induced by each stress condition contained a similar histone modification profile compared to their corresponding primary nuclei, supporting the concept that pre-existing nuclear chromatin organization influences micronuclear content (Supplementary Fig. S4a–f). The relative abundance of individual histone modifications, including H3K79me2, display limited heterogeneity when comparing stress-induced MN to spontaneously occurring MN (DMSO condition) (Supplementary Fig. S3f). HeLa cells were chosen to generate sufficient MN chromatin necessary for epiproteomics; IF was used to confirm that heterogeneity in several MN-associated histone modifications was also observed in MCF10A cells (Supplementary Fig. S3g). We conclude that MN largely retain the chromatin status present in the nucleus, with a subset of modifications modestly biased for sequestration depending on the inciting stress condition.

### Active micronuclear transcription prevents cGAS localization despite retaining H3K79me2

**Fig. 3:**
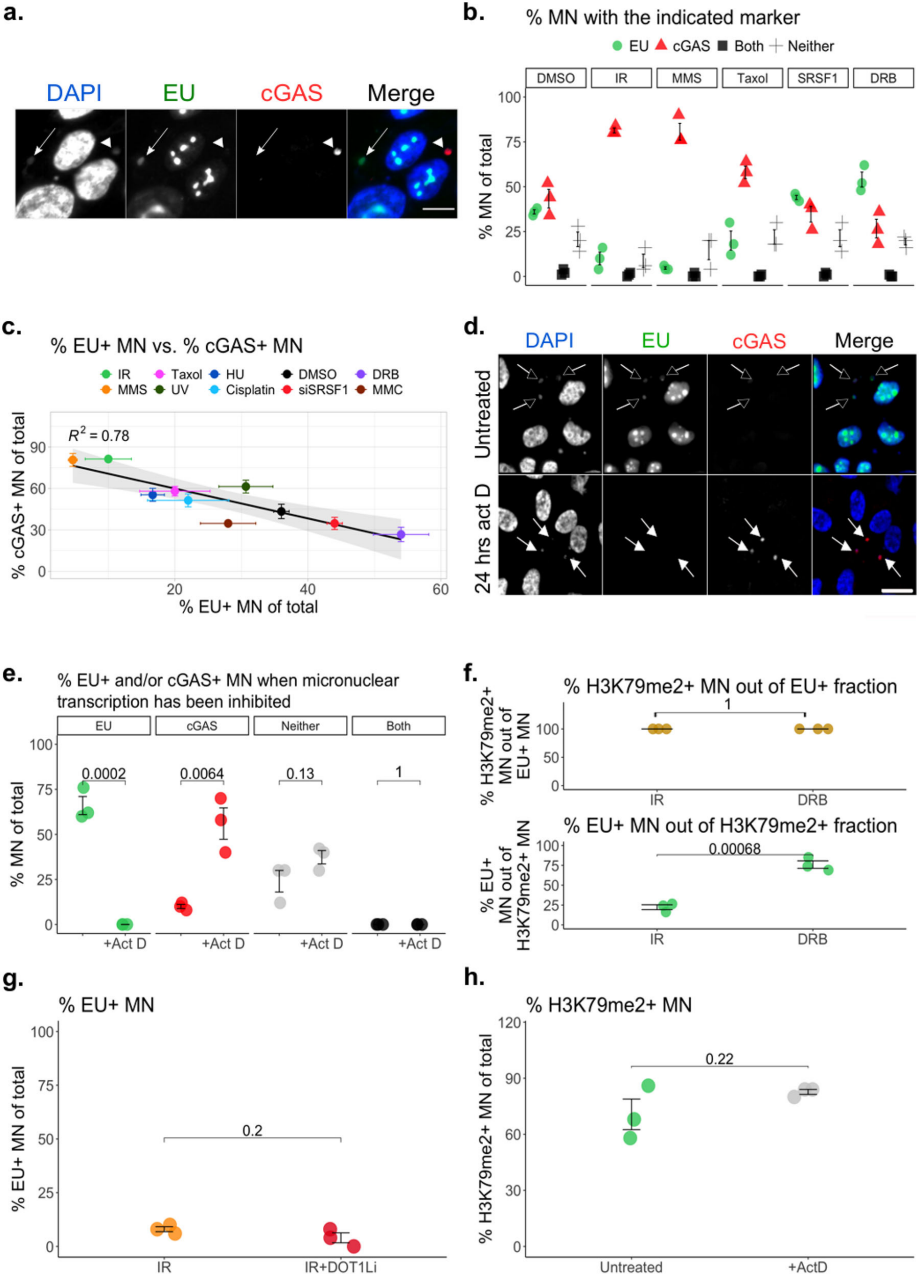
Active micronuclear transcription prevents cGAS localization despite retaining H3K79me2. **a** Representative EU+ (actively transcribing, arrow), and cGAS+ (arrowhead) MN. Scale bar = 20 μm. MCF10A cells. Images representative of 3 experiments. **b** Percent EU+ and/or cGAS+ MN by immunofluorescence (IF), 72 h post-exposure to the indicated genotoxic stressor in MCF10A. See Methods for details of each stressor. **c** Linear regression comparing pre-rupture EU+ MN to post-rupture cGAS+ MN by IF, 72 h following exposure to the indicated stressor in MCF10A. See Methods for details of each exposure. All error bars represent standard error of the mean, for 3 independent biological replicates. Vertical error bars represent cGAS+ MN, horizontal error bars represent EU+ MN. **d** Representative images of DRB-induced MN with or without actinomycin D treatment in the 24 h prior to maximal MN rupture. Empty arrows: EU+ MN; filled arrows: cGAS+ MN. Scale bar = 20 μm. MCF10A cells. Images representative of 3 experiments. **e** Percent EU+ and/or cGAS+ MN by IF, 48 h following DRB treatment. Indicated conditions were exposed to actinomycin D for 24 h prior to assessment by IF, to prevent MN transcription. MCF10A cells. **f** Percent H3K79me2+ MN out of the EU+ fraction (top) or vice versa (bottom) by IF, 72 h following exposure to the indicated stressor in MCF10A. See Methods for details of each exposure. **g** Percent EU+ MN by IF, 72 h following 10 Gy IR. MCF10A cells were pre-treated for 7 days with DOT1Li. **h** Percent H3K79me2+ DRB-induced MN with or without actinomycin D treatment in the 24 h prior to maximal MN rupture, measured by IF in MCF10A cells. All statistical comparisons performed using a two-sided Student’s *t*-test. NS: *p* = 1, ns: *p* > 0.05, *p ≤ 0.05, **p ≤ 0.01, ***p ≤ 0.001, ****p ≤ 0. All individual data points presented for immunofluorescent scoring of MN represent the mean percentage of MN that were positive for the indicated marker, from each biological replicate out of 50 total MN per replicate. All error bars represent standard error of the mean, for three independent biological replicates. Source data are provided as a Source Data file.

Our results thus far suggest that nuclear chromatin organization at the MN-destined locus affects downstream cGAS recruitment (Fig. 2a, b and Supplementary Figs. S2d–k and S3a–e). Our panel of genotoxic stress conditions generates MN with strongly divergent cGAS recruitment post-rupture (Fig. 1g), yet the total variance in histone modifications enclosed within these stress-induced MN does not show strong dissimilarity (Supplementary Fig. S3f). To resolve these observations, we considered that individual histone marks can have multifaceted functional roles, particularly when in concert with their neighboring modifications^41,45–51^. We asked whether aggregate chromatin function could be maintained on fragments that become MN-sequestered, and whether MN-contained chromatin organization affects cGAS recruitment post-rupture. As a model for an aggregate chromatin function within MN, we examined active transcription using 5-ethynyl uridine (EU) incorporation. Active MN transcription, like cGAS localization, displayed a significant association with the inciting genotoxic stress condition (Fig. 3a, b). Previous work has demonstrated that only unruptured MN are capable of transcribing^3^. Since cGAS is preferentially found within ruptured MN, at static time points cGAS displays a nearly mutually exclusive relationship with actively transcribing MN (Fig. 3b). However, we have demonstrated that the likelihood of MN attracting cGAS is not solely dependent on MN integrity (Fig. 1g). We found that, despite an equal propensity for rupture, high EU-positivity pre-MN rupture strongly predicted an inability to recruit cGAS post-rupture across our panel of stressors (Figs. 1f, g and 3c). Furthermore, we blocked active transcription by applying actinomycin D during the 24 h prior to maximal MN rupture, we significantly increased cGAS recruitment (Fig. 3d, e). These observations indicate that MN from certain stress conditions retain aggregate chromatin functions such as active transcription, and that chromatin organization within MN strongly influences cGAS localization.

The findings that H3K79me2 is a positive regulator of cGAS-MN interactions while active transcription is strongly negative were initially at odds, given the known role of H3K79me2 and me3 in marking enhancers and promoters^36,37,47^. In MN, however, we found that transcription activity was not entirely reflected by H3K79me2 status. In MN generated from either IR or DRB exposure, all actively transcribing (EU+) MN were H3K79me2+ as expected, but only a fraction of H3K79me2+ MN were transcribing (Fig. 3f). Depletion of H3K79me2 via DOT1Li did not affect the percentage of EU+ MN (Fig. 3g) and H3K79me2 levels were consistent even when transcription was blocked by actinomycin D in MN (Fig. 3h). Within MN, these data indicate that H3K79me2 can become uncoupled from ongoing transcription^47^. Since H3K79me2 has no known demethylase and relies on nucleosome turnover for its removal^35^, this decoupling may reflect an inability of MN to alter H3K79 methylation levels even as transcriptional capacity changes, with implications for cGAS chromatin engagement. Furthermore, recent reports have indicated that single-stranded (ss)DNA and RNA-DNA hybrids accumulate in MN, the latter causing chromothriptic events^19,52^. As cGAS is a dsDNA- and nucleosome-binding protein, these ssDNA- and RNA-containing structures may also contribute to reduced cGAS localization to MN that were transcribing immediately prior to rupture, even when the surrounding chromatin is H3K79-methylated^19^. Taking our observations together, we propose a model wherein cGAS-MN recognition is sensitive to aggregate chromatin context at the locus of DNA damage (Supplementary Fig. S3a–e) rather than any one individual histone modification (such as H3K79me2) that could become MN-sequestered in a biased fashion depending on the inciting genotoxic stress condition. This model is consistent with the results of our chemical screen, where the inhibition of multiple chromatin-modifying targets affected cGAS-MN localization in a genotoxic stress-dependent manner (Fig. 2a and Supplementary Fig. S2b, c). Changes to chromatin organization that occur within MN, such as activating or silencing transcription, are an additional determinant of chromatin-binding proteins such as cGAS (Fig. 3).

### The nucleosome-tethering residue of cGAS is necessary for micronuclei localization

**Fig. 4:**
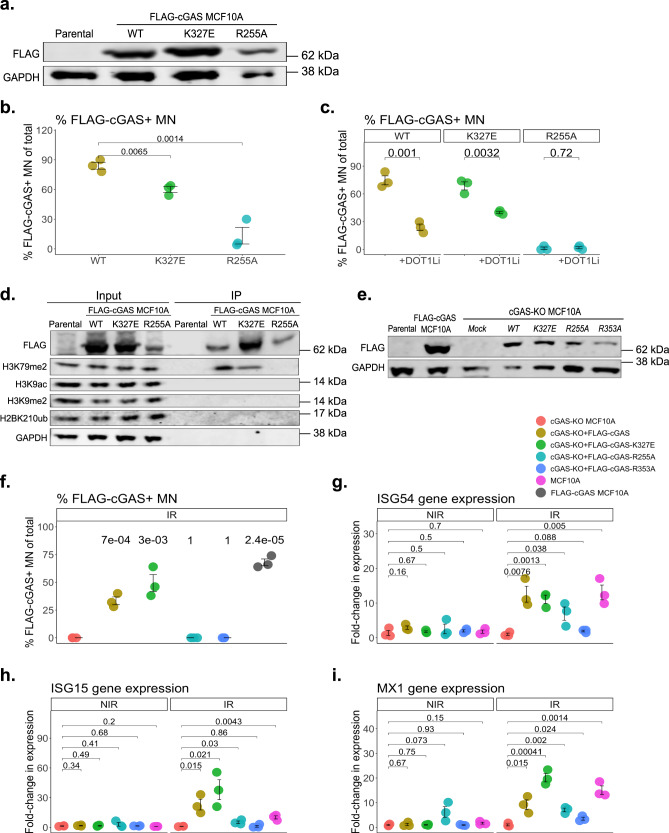
The nucleosome-tethering residue of cGAS is necessary for micronuclei localization. **a** Western blot showing stable expression of FLAG-cGAS, FLAG-cGAS^K327E^, or FLAG-cGAS^R255A^ in MCF10A cells. Image representative of 3 experiments. **b** Percent FLAG-cGAS+ MN by immunofluorescence (IF) in MCF10A cells, 72 h following 10 Gy IR. **c** Percent FLAG-cGAS+ MN by IF in MCF10A cells, 72 h following 10 Gy IR, with or without 7-day pre-treatment with DOT1Li. **d** FLAG-cGAS co-immunoprecipitated (IP) from each of the indicated cell types, 72 h following 10 Gy IR. Image representative of 3 experiments. **e** cGAS expression in cGAS-KO MCF10A cells, 72 h post-transfection with the indicated FLAG-cGAS construct. MCF10A cells stably expressing FLAG-cGAS are shown as a control. Image representative of 3 independent experiments. **f** Percent FLAG-cGAS+ MN by IF in cGAS-KO MCF10A cells, 72 h following 10 Gy IR, transfected with the indicated FLAG-cGAS construct 24 h prior to IR. Statistical comparisons by Student’s *t*-test use cGAS-KO as the reference group, where individual *p* values are shown for each comparison. **g**-**i** *ISG* expression by RT-qPCR, 72 h post-exposure to 10 Gy IR. cGAS-KO MCF10A cells were transfected with the indicated FLAG-cGAS construct 24 h prior to IR. All individual data points presented for immunofluorescent scoring of MN represent the mean percentage of MN that were positive for the indicated marker, from each biological replicate out of 50 total MN per replicate. All statistical comparisons performed using a two-sided Student’s *t*-test unless otherwise indicated. ns: *p* > 0.05, *p ≤ 0.05, **p ≤ 0.01, ***p ≤ 0.001, ****p ≤ 0. All error bars represent standard error of the mean, for three independent biological replicates. Source data are provided as a Source Data file.

We next explored possible physical mechanisms that would allow variable MN chromatin organization to affect the binding ability of cGAS. DOT1L deposits H3K79me2 on the nucleosome surface, ~37 Å from the nucleosome acidic patch, affecting local chromatin structure^41,50^. cGAS has distinct binding domains for recognizing both naked dsDNA and chromatin-associated nucleosomes, including a tethering residue for the nucleosome acidic patch at the histone 2A (H2A)-H2B dimer interface^20–24^. We speculated that changes to MN chromatin organization were impacting cGAS binding through its discrete dsDNA- and nucleosome-binding domains, providing a structural explanation for the observed sensitivity to MN chromatin status (Fig. 2a and Supplementary Fig. 2b, c). In vitro, cGAS can tether to the acidic patch via its arginine 255 (R255) residue^20,21,24^. To determine whether cGAS acidic patch tethering plays a role in MN localization, we generated MCF10A cells stably expressing either FLAG-tagged wild-type (WT) cGAS or an R255A mutant. Cells expressing a cGAS dsDNA binding site mutant (K327E) served as a control, since K327E mutants maintain their ability to tether to the acidic patch^24^ (Fig. 4a). R255A, but not K327E, reduced cGAS recruitment to MN ten-fold (Fig. 4b), and DOT1Li prevented cGAS^K327E^ from binding MN, similar to cGAS^WT^ (Fig. 4c). This pattern was mirrored when HEK293 cells, which do not express endogenous cGAS, were transfected with a FLAG-cGAS^WT^, ^-K327E^, or ^-R225A^-encoding plasmid (Supplementary Fig. S5a–c). FLAG-cGAS^WT^ and -cGAS^K327E^, but not -cGAS^R255A^, co-immunoprecipitated with H3K79me2, supporting an interaction between the R255 residue and the nucleosome where H3K79me is deposited (Fig. 4d). In the nucleus, tethering to chromatin via R255 can place cGAS in an inactive conformation^22–24^. To address whether nucleosome-tethered cGAS in MN represents an active pool, we transfected cGAS-knockout (KO) MCF10A cells with mRNA encoding FLAG-cGAS^WT^, -cGAS^K327E^, -cGAS^R255A^, or catalytically inactive FLAG-cGAS^R353A^ ^24^ (Fig. 4e, f). Only cells expressing FLAG-cGAS^R353A^ were unable to rescue a significant ISG response following IR exposure and MN formation (Fig. 4g–i). In absence of MN localization, cGAS^R255A^ induces *ISG* expression, albeit to a lesser extent than cGAS^WT^ or cGAS^K327E^, in agreement with reports that the R255A mutation can generate nucleosome-independent cGAS activity^23,24^ (Fig. 4f–i). This pattern was also observed in FLAG-cGAS-transfected HEK293 cells, under both spontaneously occurring and IR-induced MN conditions (Supplementary Fig. S5d–f) and was dependent on mitotic progression and MN formation (Supplementary Fig. S5g–i). Thus, both cGAS^WT^ and cGAS^K327E^ maintain nucleosome tethering and MN localization, and can elicit MN-dependent ISG signaling, supporting our emerging model that nucleosome-tethered cGAS is a catalytically active pool in MN. This is consistent with prior observations of active cGAS in MN using a tripartite cGAS activity reporter system^53^. We speculate that, unlike nuclear cGAS pools, increased cGAS:DNA ratios allow distinct DNA conformations within MN, creating discrete microenvironments conducive to cGAS catalytic engagement even when nucleosome-bound^4^.

Our data are consistent with the following model of micronuclear composition and activity: cGAS is sensitive to broad-scale organization of MN-enclosed chromatin fragments, which reflects the antecedent nuclear state of the MN-destined locus (Fig. 2b and Supplementary Fig. S3a–e). Distinct forms of genotoxic stress generate MN with unique chromatin functional capacity (such as active transcription), and exhibit biased cGAS recruitment (Fig. 4b). Through cGAS localization, changes to nuclear status and MN chromatin content translate into differential capacity for the cell to initiate an ISG response following unresolved DNA damage (Figs. 1h, i and 2d, e). We have demonstrated several specific deterministic features for the recruitment of cGAS to MN along the axis from nuclear chromatin status to MN envelope rupture. One such feature is histone post-translational modifications that become MN-sequestered, including DOT1L-dependent H3K79me2, but other marks are likely to prove impactful either alone or in combination (Fig. 2a and Supplementary Fig. S2b, c). Mechanistically, we found that the cGAS nucleosome acidic patch-tethering residue R255 was necessary for MN localization (Fig. 4b–d). It may be that nucleosome-local chromatin changes, such as those exerted by H3K79 methylation around the acidic patch, can influence cGAS-MN localization via its nucleosome-binding domain^41,50^. These observations further contribute to a burgeoning understanding of the relationship between cGAS tethering to nucleosomes and cGAS activity: while prior structural and in vitro studies interpreted R255-mediated nucleosome tethering as a straightforward inhibitor of cGAS activity^22–24^, more recent in vivo work has indicated that nucleosome-associated features, such as the degree of local chromatin flexibility, are dictating cGAS activity once nucleosome-bound^54^. The preservation of cGAS-dependent ISG signaling when the R255 residue is engaged in our study suggests a unique chromatin context facilitating cGAS activation in MN that is distinct from the primary nucleus (Fig. 4g–i and Supplementary Fig. S5d–f)^22–24^.

In summary, we find that the nature of the genotoxic lesions causing MN formation, by virtue of MN-sequestered chromatin organization, modulates cGAS localization and activation. Several lines of evidence presented here imply that MN-sequestered fragments inherit their chromatin characteristics from their parental nuclei (Fig. 2b and Supplementary Figs. S2b, c, 3a–e and 4). Furthermore, cGAS-dependent, locally produced interferon signaling drives immune-mediated tumor restriction in vivo^7,8^. Therefore, our new data have significant implications for the role of basal chromatin status for tumorigenesis and the response to genotoxic cancer treatments. Among cells experiencing discrete forms of genotoxic stress, we speculate that the chromatin features of MN-sequestered fragments may exhibit cell type-specificity, underlying diversity in damage consequences including inflammatory programs. Tumors or untransformed tissues with specific basal histone methylation patterns may produce more cGAS-dependent inflammatory signaling, influencing their relationship to the local immune system and their response to chemotherapeutics. DOT1L inhibitors, already in clinical trials to treat mixed-lineage leukemia^55^, could be leveraged in situations where abrogating the cGAS-driven inflammatory cascade is therapeutically desirable including certain autoimmune conditions like Aicardi–Goutieres syndrome^56^. Heterogeneity in cGAS recognition of MN has been observed since their relationship was discovered, and the lack of known molecular determinants for cGAS recruitment has complicated efforts to understand the role of MN in DNA damage-driven inflammatory programs^7–9,19^. Our findings support MN as central integrators of basal cellular characteristics and the signaling consequences of genotoxic stress.

## Methods

### Cell lines

MCF10A cells (ATCC cat #CRL-10317) were cultured in 1:1 mixture of F12:DMEM media supplemented with 5% horse serum (Wisent Bioproducts cat #098150), 20 ng/mL human EGF (Cedarlane Labs cat #AF-100-15), 0.5 mg/mL hydrocortisone (Sigma-Aldrich cat #H0888), 100 ng/mL cholera toxin (Sigma-Aldrich cat #C8052) and 10 mg/mL recombinant human insulin (SAFC cat #91077 C). HeLa-S3 (ATCC cat #CCL-2.2), U2OS (ATCC cat # HTB-96), HEK293 (ATCC cat #CRL-1573) and HEK293T (ATCC cat#CRL-3216) cells were grown in DMEM supplemented with 10% FBS. All media was supplemented with 1% penicillin-streptomycin. All cell lines were authenticated by ATCC using short tandem repeat sequencing.

### Acute treatments with varied genotoxic stressors

Treatments were performed on cultured cells. Doses and exposure times were selected based on existing literature and preliminary experiments to induce DNA lesions and maximize MN formation, while minimizing cell death and cell cycle arrest within the time frame of the assay (72 h). Doses and exposure times used for each agent are as follows: ionizing radiation (IR, Cs-137), 10 Gy^8^; methylmethanesulfonate (MMS, Sigma-Aldrich cat #12995), 0.5 μM for 4 h^57^; ultraviolet radiation (UV, 254 nm), 15 J/m^2^ ^58^; paclitaxel (Taxol, Selleck Chemicals cat #S1150), 10 nM for 4 h^59^; hydroxyurea (HU, Sigma-Aldrich cat #148627), 2 mM for 24 h^59,60^; cisplatin (Millipore cat #232120), 0.5 μg/mL for 24 h^61^; siSRSF1 (Thermo Fisher Scientific, cat #4427037, ID #s12725), 2 nM for 24 h^62^; mitomycin C (MMC, Sigma cat #M4287), 0.5 μg/mL for 4 h^6,63^; 5,6-dichloro-1-beta-ribo-furanosyl benzimidazole (DRB, Sigma-Aldrich cat #D19116), 100 μM for 4 h^62^. At the end of the designated exposure time, cells were washed in PBS, replaced with fresh media, and allowed to cycle freely for 72 h to produce MN before proceeding to downstream analysis. For all presented experiments, DMSO was used as the control for the pharmacological stressors; a non-targeting siRNA (siNT) as the control for siSRSF1, and untreated cells as the control for IR. Due to their equivalency for the relevant MN metrics (Supplementary Fig. 1b), we presented only one of these three controls in composite figures evaluating the full panel of genotoxic stressors.

### Immunofluorescent microscopy

For immunofluorescent microscopy (IF), cells were seeded onto glass coverslips 24 h prior to treatments with acute genotoxic stressors. After treatment, cells on coverslips were washed twice with PBS+ 0.1% Tween-20 (PBS-T). Cells were fixed for 10 min on ice with 3% paraformaldehyde (PFA)+ 2% sucrose in PBS. Cells were washed twice in PBS-T, then permeabilized for 10 min at room temperature with 0.5% NP-40 solution in PBS. Cells were washed twice in PBS-T, incubated in a blocking solution (3% bovine serum albumin [BSA] in PBS-T) for 10 min at room temperature. Cells were stained with the appropriate primary antibodies for a given experiment, overnight at 4 °C. The following dilutions were used for primary antibodies: 1:1000 cGAS (Cell Signalling Technologies cat #15102S); 1:200 H3K79me2 (Abcam cat #ab3594); 1:1000 FLAG (Sigma-Aldrich cat #F1804); 1:500 H3K27me3 (Cell Signalling Technologies cat #9733); 1:500 H3K4me2 (Abcam cat #32356); 1:500 H3K36me1 (New England Biolabs cat #14111S); 1:500 H3K9me3 (New England Biolabs cat #9649T); 1:500 H4K16ac (New England Biolabs cat #13534S); 1:500 H3K4me3 (New England Biolabs cat #9751S); 1:500 H4K20me1 (Abcam cat #78513). Cells were washed four times in PBS-T, then incubated in fluorescently labeled secondary antibodies for 1 h at room temperature. The following dilutions were used for secondary antibodies: 1:500 AlexaFluor 568 Goat anti-rabbit IgG secondary (Invitrogen cat #A11036); 1:500 AlexaFluor 488 Goat anti-mouse IgG secondary (Invitrogen cat #A11001); 1:500 AlexaFluor 568 Goat anti-mouse IgG secondary (Invitrogen cat #A11004); 1:500 AlexaFluor 488 Goat anti-rabbit IgG secondary (Invitrogen cat #A11034); Cells were washed four times in PBS-T, then inverted onto a drop of Prolong Glass Antifade Mountant with NucBlue (DAPI) stain (Thermo Fisher Scientific, cat #P36981) on a microscope slide. Slides were imaged on an Olympus immunofluorescent microscope, using CellSens Dimensions software. All IF images presented here were taken at 60× objective on an oil lens. All IF experiments evaluating a percentage of marker-positive MN counted 50 MN per biological replicate.

### Live cell imaging

Live cell imaging of MCF10A cells stably expressing GFP-tagged nuclear localization signal (GFP-NLS), mCherry-tagged cGAS (mCherry-cGAS), or mCherry-tagged histone 2B (mCherry-H2B) were performed using an Incucyte SX5 (Sartorius Imaging). Images were taken once every 60 min to acquire videos of cells monitored over a 72-h period.

### Preparation of cGAS binding mutant plasmids

Site-directed mutagenesis following the New England Biolabs Q5 Site-Directed Mutagenesis kit (cat #E0554) using pOZ-FLAG-cGAS (cloned as previously described^8^) as the template to generate plasmids containing point mutations for cGAS^K327E^, cGAS^R255A^, and cGAS^R353A^. Stable MCF10A lines with viral infection were generated as described below.

### Viral infection and selection of stable cell lines

To prepare MCF10A and HeLa cells stably expressing GFP-NLS, FLAG-cGAS (and mutants), mCherry-cGAS, and/or mCherry-H2B, lentiviral transfection followed by fluorescence-activated cell sorting (FACS) or antibiotic selection was performed. pTRIP-SFFV-EGFP-NLS (Addgene ID #86677), pOZ-cGAS (puromycin-resistant, cloned as previously decribed^8^), pLXV-mCherry-cGAS (puromycin-resistant cloned as previously decribed^8^), and/or pLenti6-mCherry-H2B (Addgene ID #89766, blasticidin-resistant) were transfected into HEK293T cells as follows, according to LipoD293 reagent instructions (FroggaBio cat #SL100668.1): Lentiviral packaging plasmids pMD2.g (Addgene #12269), pRSV-Rev (Addgene #12253), and PMDLg/pRRE (Addgene #12251) were combined in serum-free DMEM, using 0.5 μg of each plasmid for 125 μL of DMEM. 1 μg of target plasmid was added to this mixture per 125 μL DMEM. In a separate tube, 7.5 μL of LipoD293 was added per 125 μL DMEM. The two mixtures were combined and allowed to incubate at room temperature for 30 min, then added dropwise to HEK293T cells cultured in DMEM supplemented with 10% FBS and 1% penicillin-streptomycin. Cell supernatants containing lentiviruses were mixed 1:1 with target cell media and supplemented with 4 mg/mL polybrene (Sigma-Aldrich cat #107689). Successfully transduced cells were selected using puromycin (Cedarlane cat #13884-50), blasticidin (Cedarlane cat #14499-25), or in the case of GFP-NLS-infected cells, FACS sorting. pOZ-cGAS and pLXV-mCherry-cGAS plasmids were cloned as previously described^8^.

### shRNA infections

TRC pLKOpuro lenti plasmids encoding shDOT1L (CCCGGATCTCAAGCTCGCTAT) or shLuciferase (ACGCTGAGTACTTCGAAATGT) were given to us by the Structural Genomics Consortium (SGC) and packaged into lentiviral vectors as described above. HEK293T cells supernatants containing lentiviruses were mixed 1:1 with target cell (MCF10A) media and supplemented with 4 mg/mL polybrene (Sigma-Aldrich cat #107689). Media was changed 48 h after infection and replaced with whole-growth media. Infection was repeated on day 0, day 4, and day 7.

### siRNA transfections

siRNA were transfected using the Lipofectamine RNAiMAX Transfection Reagent from Thermo Fisher (cat #13778075) according to the manufacturer’s instructions. All siRNAs were purchased from the Thermo Fisher Silencer Select siRNA catalog, with the following catalog numbers: non-targeting siRNA (siNT) catalog #4390843; siSRSF1 (1) catalog #4427037 ID #s12727; siSRSF1 (2) catalog #4427037 ID #s12725; siRNF20 (1) catalog #4392420 ID #s32087; siRNF20 (2) catalog #AM16708 ID #132879.

### Plasmid transfections

HEK293 cells were transfected with plasmids encoding pOZ-cGAS (wild-type or mutant, generated as described above) using the LipoD293 transfection reagent (SignaGen, cat # SL100668) according to the manufacturer’s instructions.

### Transfecting cGAS-KO MCF10A cells with mRNA for cGAS binding mutant transgenes

The plasmids encoding cGAS mutants cGAS^K327E^, cGAS^R255A^, and cGAS^R353A^ (generated as described above) were reverse-transcribed into mRNA using the mMessage mMACHINE™ T7 ULTRA Transcription Kit (Thermo Fisher Scientific, cat #AM1345). MCF10A cells were transiently transfected with the mRNA using Lipofectamine Messengermax (Invitrogen cat #LMRNA001). All instructions associated with both kit protocols were followed as prescribed.

### cGAMP ELISA

cGAMP production was measured using the 2’3’-cGAMP ELISA kit from Cayman Chemicals (cat # 501700-96) according to the manufacturer’s instructions.

### Western blotting

Cells were harvested by scraping and pelleted by centrifugation at 500 g for 5 min. Cell pellets were resuspended in a lysis buffer composed of RIPA buffer freshly supplemented with 1 mM benzamidine HCl, 1 μg/mL antipain, 5 μg/mL aprotinin, 1 μg/mL leupeptin, 0.5 mM phenylmethylsulfonyl fluoride (PMSF), 1 mM DTT, 2 mM NaOV_4_, 10 mM NaF, 2 mM imidazole, 1.15 mM sodium molybdate, and 4 mM sodium tartrate (Sigma). Lysis was conducted on ice for 20 min, vortexing cells briefly after 10 min to prevent them from settling. Lysate was centrifuged at 18,000 g for 15 min at 4 °C, and the supernatant was transferred to a clean microcentrifuge tube. Protein amount was measured by Bradford assay, and 20 μg of protein was diluted to 20 μL in ddH_2_O, then combined with 5 μL of 5× sample buffer (50 mM Tris, 10% glycerol, 2% SDS, 0.01% bromophenol blue, 2.5% β-mercaptoethanol), denatured at 95 °C for 10 min, and resolved on a Bolt™4–12% Bis-Tris Plus Gel (Thermo Fisher Scientific cat #NW04127BOX). Proteins were transferred from the gel to a nitrocellulose membrane (LI-COR Biosciences cat #926-31092). Membranes were blocked in 5% milk in PBS-T for 1 h and incubated with primary antibody overnight at 4 °C. Membranes were then washed 4 times in PBS-T and incubated for 1 h at room temperature with fluorescently conjugated secondary antibodies diluted 1:20,000. The following primary antibodies were used for western blots: 1:1000 cGAS (Cell Signalling Technologies cat #15102S); 1:500 H3K79me2 (Abcam cat #ab3594), 1:500 H2BK120ub (Cell Signalling Technologies cat #5546T); 1:500 H3K9ac (Cell Signalling Technologies cat #9649T); 1:500 H3K9me2 (Abcam cat #ab32521); 1:1000 Total Histone H3 (Abcam cat #1791); 1:1000 FLAG (Sigma-Aldrich cat #F1804); 1:1000 GAPDH (New England Biolabs cat #97166S); SRSF1 (Thermo Fisher cat #324500);. The following secondary antibodies were used for western blots: Donkey Anti-Rabbit IgG AlexaFluor 790 (Jackson ImmunoResearch Labs cat #711-655-152), Donkey Anti-Mouse IgG AlexaFluor 680 (Jackson ImmunoResearch Labs cat #711-655-152). Following four washes in PBS-T, membranes were imaged using a LI-COR. All displayed western blots are representative of at least three independent experiments.

### Gene expression analysis

Total RNA was isolated from cells using TriZol reagent (Life Technologies cat #15596018) according to the manufacturer’s instructions. cDNA was generated from 1000 ng of RNA using the iScript cDNA synthesis kit (Bio-Rad cat #1708891) according to the manufacturer’s instructions. qPCR was performed with gene-specific primers using SSO Advanced Universal SYBR Green Supermix (Bio-Rad cat #1725274) on a Bio-Rad C1000Touch Thermocycle. Relative transcription levels were calculated by normalizing to *GAPDH* expression. The following gene-specific primers were used: *cGAS* Forward (AGGAAGCAACTACGACTAAAGCC) and Reverse (CGATGTGAGAGAAGGATAGCCG); *GAPDH* Forward (CTCAAGATCATCAGCAATGCC) and Reverse (CATCACGCCACAGTTTCCC); *ISG54* Forward (AAGCACCTCAAAGGGCAAAAC) and Reverse (CTCTGAGCATCCTGGTGAGGAA); *ISG15* Forward (CTCTGAGCATCCTGGTGAGGAA) and Reverse (AAGGTCAGCCAGAACAGGTCGT); *MX1* Forward (GGCTGTTTACCAGACTCCGACA) and Reverse (CACAAAGCCTGGCAGCTCTCTA); *STING* Forward (CCTGAGTCTCAGAACAACTGCC) and Reverse (GGTCTTCAAGCTGCCCACAGTA).

### HT-DNA or cGAMP pre-treatment to induce ISG

To generate positive control conditions for *ISG* qPCR experiments presented in Fig. 2d, e, MCF10A cells were either transfected with 1 μg of herring testes (HT)-DNA using the jetPRIME transfection reagent from Polyplus (cat #101000027) or were exposed to 100 μg/mL 2’−3’-cGAMP from Invivogen (cat #tlrl-nacga23-5) added directly to the media. HT-DNA transfection was performed 24 h prior to IR exposure (transfection media replaced after 4 h), according to jetPRIME manufacturer’s instructions. cGAMP was added to cells 24 h prior to IR exposure and remained for the duration of the experiment.

### Immunoprecipitation

MCF10A or HeLa cells expressing FLAG-tagged cGAS were harvested by scraping and pelleted by centrifugation at 3000 g for 10 min at 4 °C. The cell pellet was resuspended in NET-N lysis buffer (100 nM NaCl, 50 mM Tris-HCl pH 8.0, 2 mM EDTA, 0.5% NP-40, and 10 nM MgCl_2_) freshly supplemented with 10 mM NaF, 2 mM imidazole, 1.15 mM sodium molybdate, and 4 mM sodium tartrate, 200 mM sodium butyrate, 10 units/mL benzonase, 1 mM DTT, 0.5 mM PMSF, 1 mM benzamidine HCl, 1 μg/mL antipain, 5 μg/mL aprotinin, and 1 μg/mL leupeptin (Sigma). Cells were left in lysis buffer on ice for 30 min, vortexing briefly every 10 min to prevent the cells from settling. Lysate was centrifuged at 18,000 g for 15 min at 4 °C, and the supernatant was transferred to a clean microcentrifuge tube. Protein amount was measured with a Bradford assay. 10 μg of protein was kept as input, and 500–1000 μg of protein was taken for immunoprecipitation (IP). Protein for IP was diluted in NET-N buffer to a total volume of 500–1000 μL. FLAG-M2 beads (Sigma-Aldrich cat #A2220) were washed once in PBS and once in NET-N buffer, centrifuging 1000 g for 3 min at 4 °C to pellet the beads. Equilibrated FLAG-M2 beads were resuspended in NET-N buffer, and 10 μL beads were added to 1000 μL of IP protein solution. The microcentrifuge tubes containing the IP protein extract + FLAG-M2 beads were left at constant rotation overnight at 4 °C. The beads were pelleted by centrifugation at 1000 g for 3 min at 4 °C, and the supernatant was saved as flow-through. The beads were washed four times in 1 mL NET-N buffer, centrifuging at 1000 g for 3 min at 4 °C. The immunoprecipitate was then eluted from the beads by competition with FLAG peptide (Sigma-Aldrich cat #F47799) as follows: pelleted beads were resuspended with 20 μL of 0.2 mg/mL FLAG peptide per 10 μL beads and left to incubate for 1 h at 4 °C. The mixture was redistributed by pipetting every 15 min. At the end of the incubation, beads were pelleted by centrifugation at 1000 g for 3 min at 4 °C, and the supernatant containing eluted FLAG-cGAS was moved to a fresh microcentrifuge tube. At this point, protein samples were used for western blotting as described above. All displayed immunoprecipitation blots are representative of at least three independent experiments.

### Epigenetic inhibitor screen

The screen presented in Supplementary Fig. S2d–j was performed using a library of chemical inhibitors, each designed to inhibit a specific histone reader, writer, or eraser^31,32^. The screen takes place in a 96-well plate containing 100× stock solutions of each inhibitor, with concentrations chosen according to the screen manufacturer’s specifications (Supplementary Table S1). MCF10A cells were seeded in 96-well plates in 198 μL of growth media, 24 h prior to the start of the screen. They were seeded in a Corning high-content imaging plate (cat #CLS4580), allowing for IF microscopy to take place directly within the wells. 2 μL of each 100× inhibitor were added to the wells containing MCF10A cells, diluting to 1×. Cells were treated with the library for 4 days, as this is indicated by the screen’s specifications to be sufficient time for all targeted histone modifications to be affected. After 4 days, the plate was exposed to 10 Gy IR, and left for another 72 h to produce MN while still in the presence of the epigenetic inhibitors. 72 h post-IR exposure, the inhibitors were washed off. Fixation, permeabilization, blocking, and primary/secondary antibody staining were conducted as described above for IF microscopy, but on cells at the bottom of the imaging plates rather than on glass coverslips. After antibody staining, wells were filled with 150 μL of 300 nM DAPI (Life Technologies cat #D1306) in PBS, and imaged on an Olympus immunofluorescent microscope as described for IF microscopy.

### Chronic pre-treatment with DOT1Li for IF evaluations of cGAS+ MN

MCF10A cells were seeded onto glass coverslips in 24-well plates, at very low density (10,000 cells per well) and allowed to settle overnight. Cells were then treated with 1 μM SGC0946, 2 μM EPZ5676, or 5 μM EPZ4777 for 7 days. Cells were exposed to 10 Gy IR, and allowed to cycle for an additional 72 h, still in the presence of the DOT1Li. Cells were then processed for immunofluorescent imaging, as described above. Cells are not split during this 10-day time frame; seeding density should be low enough that cells do not reach confluency prior to IR exposure.

### Histone acid extraction

For the immunoblots presented in Fig. 2b, histones were acid-extracted from lysates prior to loading, following a previously described protocol^64^. Treated cells were harvested by scraping and pelleted by centrifugation at 500 g for 5 min. Cell pellets were resuspended in 200 μL nuclear isolation buffer (NIB; 15 mM Tris-HCl, 60 mM KCl, 15 mM NaCl, 5 mM MgCl_2_, 1 mM CaCl_2_, 250 mM sucrose, pH adjusted to 7.5) freshly supplemented with 0.5 mM DTT, 1 mM PMSF, 10 mM NaF, 2 mM imidazole, 1.15 mM sodium molybdate, 4 mM sodium tartrate, and 200 mM sodium butyrate (Sigma). Cells were centrifuged at 1000 g for 5 min at 4 °C, and the supernatant was discarded. Cell pellet was resuspended in freshly supplemented NIB+ 0.2% NP-40 for lysis, and incubated on ice for 15 min. Cells were vortexed briefly after 5 min, to prevent them from settling. After lysis, cells were pelleted by centrifugation at 1000 g for 5 min at 4 °C, and the supernatant was discarded. Cells were washed twice in supplemented NIB. After the second wash, cell pellet was resuspended in 400 μL chilled 0.2 M H_2_SO_4_, and incubated for three h at constant rotation. The extraction was then centrifuged at 11,000 g for 5 min at 4 °C, and the supernatant was transferred to a fresh microcentrifuge tube. 132 μL of 100% tricholoracetic acid (Sigma-Aldrich cat #T9159) was added dropwise to the supernatant, to a final concentration of 33%. The tubes were mixed by inverting, then incubated at 4 °C overnight. Cells were then pelleted by centrifugation at 16,000 g for 10 min at 4 °C, and the supernatant was discarded carefully, without scraping the sides or the bottom of the tube. The sides and bottom of the tube were rinsed with 1 mL of ice-cold 0.1% HCl in acetone, centrifuging 16,000 g for 10 min at 4 °C and carefully discarding the supernatant without scraping the sides or the bottom of the tube. The ΗCl wash was repeated once, then when the supernatant was carefully discarded the tubes were left open at room temperature for 20 min to dry. The histones coating the sides and bottom of the tube were resuspended in 20–40 μL of ddH_2_O, and at this point were used for western blotting as described above.

### Subcellular fractionation/micronuclei purification

Micronuclei and nuclei were purified prior to epiproteomic mass spectrometry according to a previously described protocol^9^. 500 mL suspension-cultured HeLa cells pre-treated with acute genotoxic stressors were harvested and washed twice in DMEM without serum. Cells were resuspended in DMEM without serum, supplemented with cytochalasin B (BioShop Canada cat #CYT444.25) at 10 μg/mL, and incubated at 37 °C for 30 min. Cells were centrifuged at 250 × g for 5 min and the cell pellet was resuspended in ice-cold lysis buffer (10 mM Tris-HCl pH 8.0, 0.32 mM sucrose, 2 mM magnesium acetate, 3 mM calcium chloride, 0.1 mM EDTA, 0.1% NP-40, pH adjusted to 8.5) freshly supplemented with 10 μg/mL cytochalasin B, 0.15 mM spermine, 0.75 mM spermidine, 1 mM dithiothreitol (DTT) and 200 mM sodium butyrate (Sigma). Resuspended cells were homogenized by douncing, using 15 strokes with a loose-fitting pestle. Cell lysates were then mixed with an equal volume of ice-cold 1.8 M sucrose buffer (10 mM Tris-HCl pH 8.0, 1.8 M sucrose, 5 mM magnesium acetate, 0.1 mM EDTA, 0.3% BSA, pH adjusted to 8.0) freshly supplemented with spermine, spermidine, DTT, and sodium butyrate. 10 mL of this mixture was layered onto a two-layer sucrose gradient, prepared by adding 20 mL of 1.8 M sucrose buffer on top of 15 mL of 1.6 M sucrose buffer in a 50 mL conical tube. The layers are added to each slowly, such that the middle 1.8 M buffer layer does not mix with the lower 1.6 M buffer layer, and the upper 1:1 mixture of lysed cells + 1.8 M does not mix with the 1.8 M buffer layer. The gradient was centrifuged for 1000 g for 20 min at 4 °C, during which time the nuclei and micronuclei contained within the lysed mixture will distribute within the gradient according to size. Fractions can be generally collected as follows: upper 3 mL contains debris and is discarded, the next 6–12 mL contains MN with minimal contaminating nuclei and is collected, final 35 mL contains primary nuclei and is not used. MN- and contaminating nuclei-containing fractions were layered on top of a 5 mL cushion of 1.2 M sucrose buffer, in a 50 mL ultracentrifuge tube (Beckman Coulter cat #344058). MN were pelleted by ultracentrifugation in an Optima XPN-80 ultracentrifuge (Beckman Coulter) at 35,000 g for 90 min in an SW41 rotor, at 4 °C. The supernatant was discarded and the pellet was resuspended in 500 μL of 1.0 M sucrose buffer. The pellet was layered on top of an 11 mL linear 1.0–1.8 M sucrose buffer gradient, prepared in a 15 mL Falcon tube as follows: Add 2.75 mL of 1.8 M sucrose buffer to the bottom of the tube. Freeze on liquid nitrogen or by brief storage at −80 °C. Layer 2.75 mL of a 2:1 mixture of 1.8 M: 1.0 M sucrose buffer onto the frozen layer. Freeze new layer as before. Layer 2.75 mL of a 1: 2 mixture of 1.8 M: 1.0 M sucrose buffer onto the frozen layer. Freeze new layer as before. Layer 2.75 mL of 1.0 M sucrose buffer onto the frozen layer. Freeze new layer as before. Thaw entire gradient upright at 4 °C when ready for use. MN pellet was centrifuged through the 11 mL linear gradient at 530 g for 15 min at 4 °C. Fractions can be generally collected as follows: upper 1–4 mL contains pure MN, the final 8 mL contains primary nuclei and fractions are monitored by DAPI staining.

### Epiproteomic mass spectrometry

MN and nuclei were fractionated as described above and sent to Northwestern Proteomics for assessment using their commercial Epiproteomic Histone Modification Profile [http://proteomics.northwestern.edu/epiproteomic-histone-modification-panel/].

### 5-Ethynyl uridine (EU) staining

For IF visualization of active transcription, EU staining was carried out using a click-chemistry reaction (Invitrogen cat #C10269) was performed. Staining was carried out according to click chemistry kit manufacturer’s instructions. In brief: 0.5 mM EU was added to cultured cells, 1 h prior to fixation for IF. Cells were fixed and permeabilized as described for immunofluorescent microscopy. Cells were washed twice in PBS-T, then incubated with the click chemistry reaction mixture containing an AlexaFluor 488 reactive azide (Thermo Fisher Scientific, cat #A10266) for 30 min at room temperature. Cells were washed twice in PBS-T, then processed as described for immunofluorescent microscopy, beginning from blocking with 3% BSA in PBS-T.

### Micronuclear transcription inhibition

MCF10A cells were exposed to a 4-h treatment with 100 μM DRB (Sigma-Aldrich cat #D19116) and given 24 h following washout to produce MN. Actinomycin D (0.01 μg/mL; Sigma-Aldrich cat #A9415) was then added for an additional 24 h. Since most MN have ruptured by 25 h post-formation (Supplementary Fig. S1j) and take approximately 20 h following acute DRB treatment to form, this treatment regimen inhibits MN transcription in the hours immediately prior to their rupture, whereupon changes to cGAS recruitment were assessed by immunofluorescence.

### Doxycycline-inducible transcription and directed DNA damage in U2OS cells

U2OS cells stably expressing FokI endonuclease and the 263 constructs as previously described were gifted by Dr Roger Greenberg’s lab^43,44^. Treatment with 4-hydroxytamoxifen (4-OHT, Sigma-Aldrich cat #H7904) and Shield-1 (Cheminpharma cat #CIP-S1-0005) stabilizes the endonuclease and allows its nuclear transduction, where it will induce DSBs at the targeted fusion protein^43,65^. 265-expressing U2OS cells were seeded on glass coverslips 24 h prior to treatment with 2 μg/mL doxycycline (DOX, Sigma-Aldrich cat #D3072). DOX was left overnight to drive transcription at the reporter locus. Shield-1 and 4-OHT were then added to the cells at 1 and 2 μM, respectively. Six hours later, SHIELD/OHT were washed off the cultured cells, and DOX was replaced. Cells were left for 72 h to cycle freely and generate micronuclei, before either evaluating % cGAS+ MN by IF or verifying MN content with DNA FISH.

### DNA fluorescence in situ hybridization (FISH)

Cells were seeded on glass coverslips 24 h prior to beginning FISH protocol. Cells on coverslips were washed three times in PBS, then fixed for 10 min at 4 °C with 2% paraformaldehyde in PBS. Cells were washed three times in PBS, then permeabilized for 10 min at 4 °C with 0.5% NP-40 solution. Cells were washed three times in PBS, then denatured in 50% formamide/2× sodium saline citrate (SSC) at 75 °C for 30 min. Cells were washed three times in PBS, then inverted onto a 5 μL droplet of hybridization buffer on a glass microscope slide. Hybridization buffer is composed of 50% formamide, 50% dextran, 20% SDS, 20X SSC, and 1 μL DNA FISH probe (CCA CTC CCT ATC AGT GAT AGA GAA AAG, tagged at the 3’ end with digoxigenin) per 5 μL hybridization buffer. Coverslips were sealed with CytoBond (SciGene cat #2020-00-2) and the slide was incubated at 75 °C for 10 min, then transferred to a humidified chamber to incubate at 37 °C overnight. CytoBond was then removed, and coverslips were lifted from the slide and submerged in 2× SSC. Cells on coverslips were rinsed once in 2× SSC, then washed three times with 50% formamide/2× SSC, incubating the coverslips at 37 °C for 5 min between each wash. Cells were rinsed once in PBS-T, then incubated in 3% BSA in PBS-T (blocking buffer) for 30 min at room temperature. After blocking, cells were incubated with an anti-DIG FITC-tagged probe (Millipore cat #1120774191) diluted 1:200 in a blocking buffer. Cells were incubated with the probe for 1 h at room temperature, then washed three times in PBS-T. Coverslips were then inverted onto a drop of Prolong Glass Antifade Mountant with NucBlue (DAPI) stain (Thermo Fisher Scientific, cat #P36981) on a microscope slide. Slides were imaged as described above for immunofluorescent microscopy.

### Statistical analysis

Information regarding biological replicates, sample size, and statistical testing is supplied in figure legends.

## Supplementary information

Supplementary information

## Data Availability

The epiproteomics datasets presented in Supplementary Figs. S3f and S4 are available in the online repository MassIVE, under the dataset ID MSV000089836 [https://massive.ucsd.edu/ProteoSAFe/static/massive.jsp]. The authors declare that all other data supporting the findings of this study are available within the paper and its supplementary files, and that all. Source Data are provided with this paper.

## Source data

Source data
