# Supplementary material for: Antecedent chromatin organization determines cGAS recruitment to ruptured micronuclei
Source: Nat Commun. 2023 Feb 2;14:556. doi: 10.1038/s41467-023-36195-8 (PMC9894866; doi:10.1038/s41467-023-36195-8)
Supplement: Supplementary file 1 — Supplementary information [file 41467_2023_36195_MOESM1_ESM.pdf]

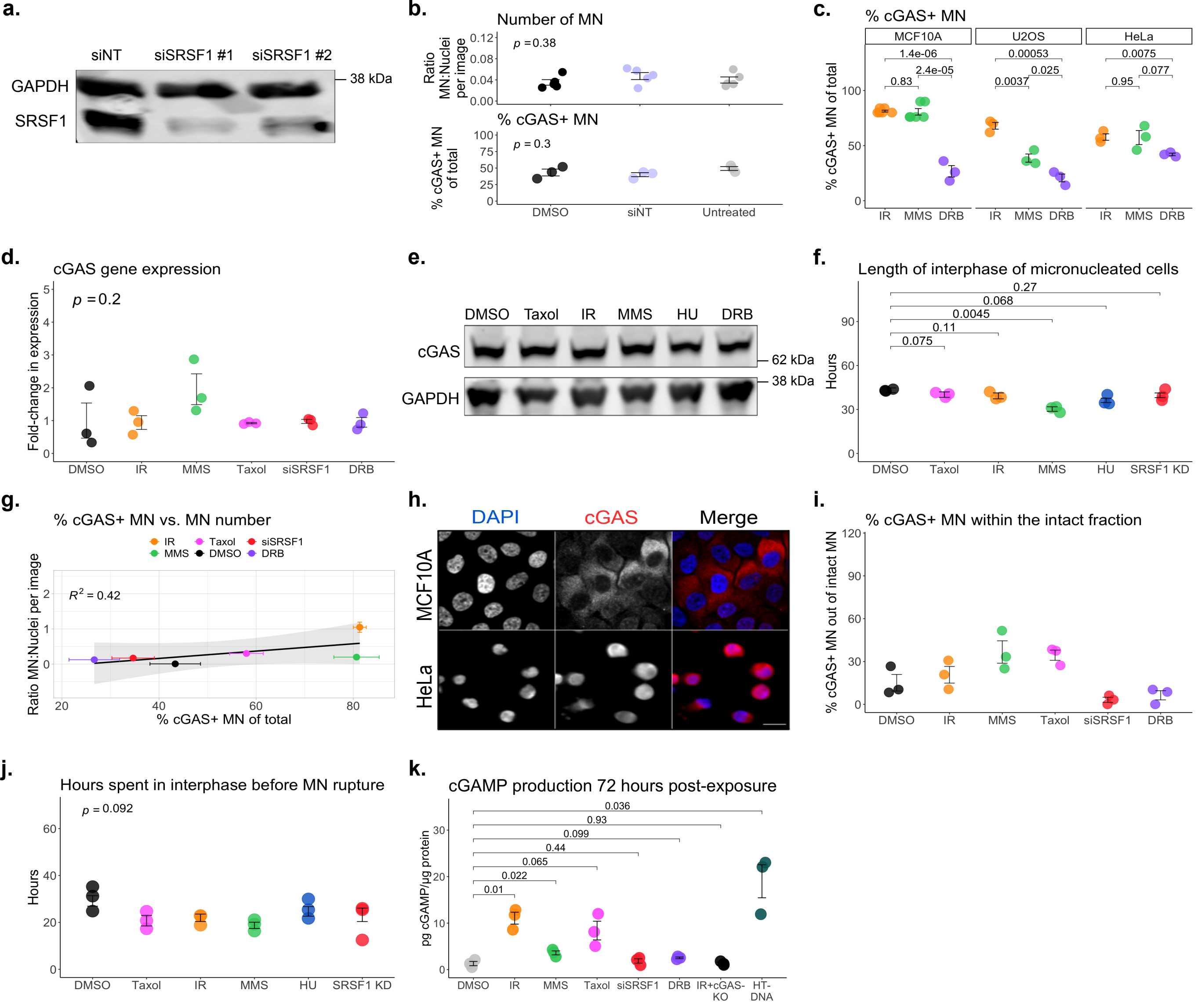
a.
siNT
siSRSF1 #1
siSRSF1 #2
GAPDH
SRSF1
38 kDa
b.
Number of MN
Ratio MN:Nuclei per image
p = 0.38
% cGAS+ MN
% cGAS+ MN of total
p = 0.3
DMSO
siNT
Untreated
c.
% cGAS+ MN
MCF10A
U2OS
HeLa
1.4e-06
2.4e-05
0.83
0.00053
0.025
0.0037
0.0075
0.077
0.95
% cGAS+ MN of total
IR
MMS
DRB
d.
cGAS gene expression
p = 0.2
Fold-change in expression
DMSO
IR
MMS
Taxol
siSRSF1
DRB
e.
DMSO
Taxol
IR
MMS
HU
DRB
cGAS
GAPDH
62 kDa
38 kDa
f.
Length of interphase of micronucleated cells
0.27
0.068
0.0045
0.11
0.075
Hours
DMSO
Taxol
IR
MMS
HU
SRSF1 KD
g.
% cGAS+ MN vs. MN number
IR
Taxol
siSRSF1
MMS
DMSO
DRB
R2 = 0.42
Ratio MN:Nuclei per image
% cGAS+ MN of total
h.
DAPI
cGAS
Merge
MCF10A
HeLa
i.
% cGAS+ MN within the intact fraction
% cGAS+ MN out of intact MN
DMSO
IR
MMS
Taxol
siSRSF1
DRB
j.
Hours spent in interphase before MN rupture
p = 0.092
Hours
DMSO
Taxol
IR
MMS
HU
SRSF1 KD
k.
cGAMP production 72 hours post-exposure
0.036
0.93
0.099
0.44
0.065
0.022
0.01
pg cGAMP/µg protein
DMSO
IR
MMS
Taxol
siSRSF1
DRB
IR+cGAS-KO
HT-DNA

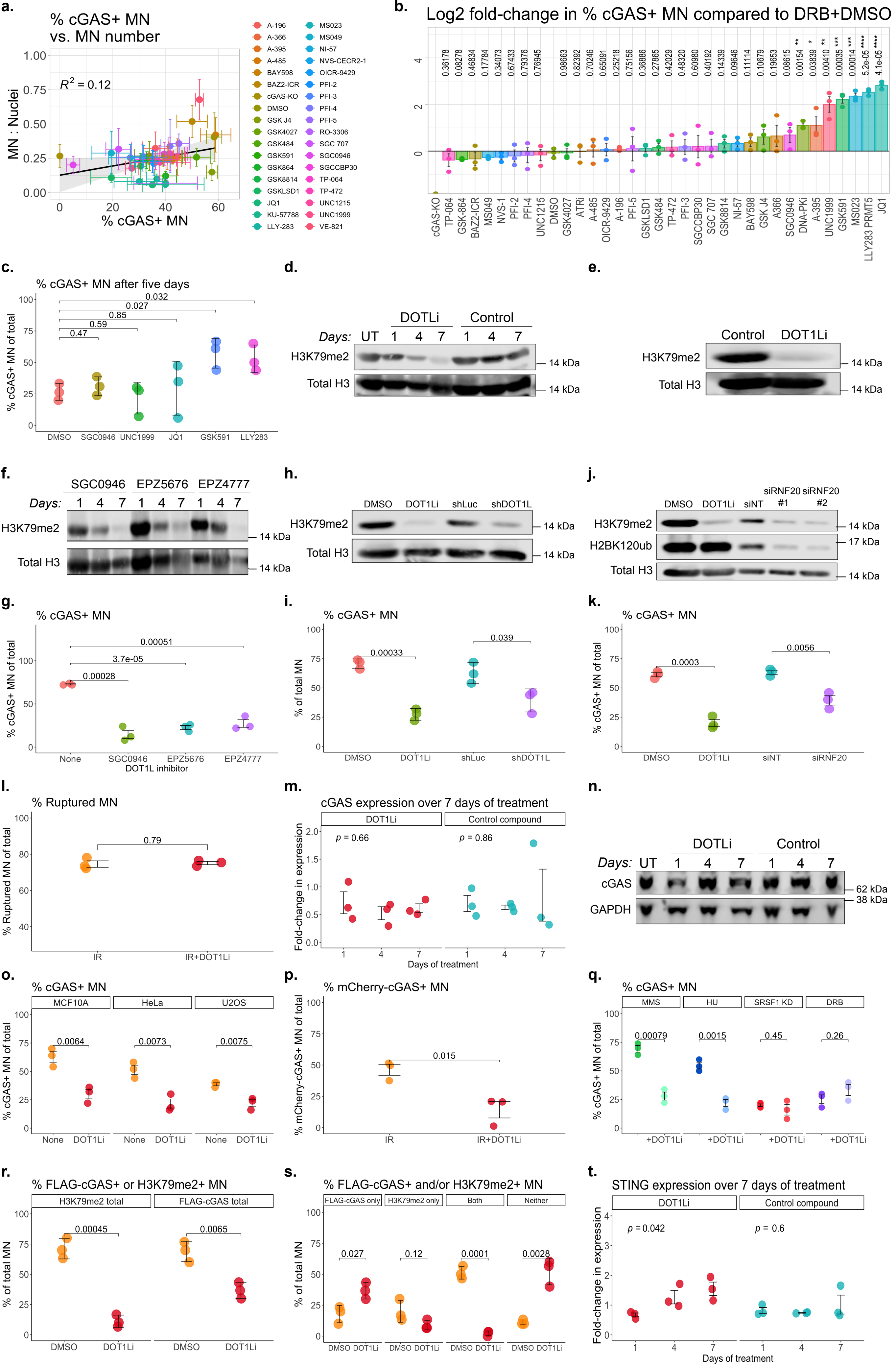
a.
% cGAS+ MN vs. MN number
R^2 = 0.12
MN : Nuclei
% cGAS+ MN
A-196
A-366
A-395
A-485
BAY598
BAZ2-ICR
cGAS-KO
DMSO
GSK J4
GSK4027
GSK484
GSK591
GSK864
GSK8814
GSKLSD1
JQ1
KU-57788
LLY-283
MS023
MS049
NI-57
NVS-CECR2-1
OICR-9429
PFI-2
PFI-3
PFI-4
PFI-5
RO-3306
SGC 707
SGC0946
SGCCBP30
TP-064
TP-472
UNC1215
UNC1999
VE-821
b.
Log2 fold-change in % cGAS+ MN compared to DRB+DMSO
0.36178
0.08278
0.46834
0.17784
0.34073
0.67433
0.79376
0.76945
0.98863
0.82392
0.70246
0.65091
0.25218
0.75156
0.36886
0.27865
0.42029
0.48320
0.60980
0.40192
0.14339
0.09646
0.11114
0.10679
0.19653
0.08615
0.00154 **
0.03839 *
0.00410 **
0.00035 ***
0.00014 ***
5.2e-05 ****
4.1e-05 ****
cGAS-KO
TP-064
GSK-864
BAZ2-ICR
MS049
NVS-1
PFI-2
PFI-4
UNC1215
DMSO
GSK4027
ATRi
A-485
OICR-9429
A-196
PFI-5
GSKLSD1
GSK484
TP-472
PFI-3
SGCCBP30
SGC 707
GSK8814
NI-57
BAY598
GSK J4
A366
SGC0946
DNA-PKi
A-395
UNC1999
GSK591
MS023
LLY283 PRMT5
JQ1
c.
% cGAS+ MN after five days
% cGAS+ MN of total
0.032
0.027
0.85
0.59
0.47
DMSO
SGC0946
UNC1999
JQ1
GSK591
LLY283
d.
DOTLi
Control
Days: UT 1 4 7 1 4 7
H3K79me2
Total H3
14 kDa
14 kDa
e.
Control
DOT1Li
H3K79me2
Total H3
14 kDa
14 kDa
f.
SGC0946
EPZ5676
EPZ4777
Days: 1 4 7 1 4 7 1 4 7
H3K79me2
Total H3
14 kDa
14 kDa
h.
DMSO
DOT1Li
shLuc
shDOT1L
H3K79me2
Total H3
14 kDa
14 kDa
j.
DMSO
DOT1Li
siNT
siRNF20 #1
siRNF20 #2
H3K79me2
H2BK120ub
Total H3
14 kDa
17 kDa
14 kDa
g.
% cGAS+ MN
% cGAS+ MN of total
0.00051
3.7e-05
0.00028
None
SGC0946
EPZ5676
EPZ4777
DOT1L inhibitor
i.
% cGAS+ MN
% of total MN
0.00033
0.039
DMSO
DOT1Li
shLuc
shDOT1L
k.
% cGAS+ MN
% cGAS+ MN of total
0.0003
0.0056
DMSO
DOT1Li
siNT
siRNF20
l.
% Ruptured MN
% Ruptured MN of total
0.79
IR
IR+DOT1Li
m.
cGAS expression over 7 days of treatment
DOT1Li
Control compound
p = 0.66
p = 0.86
Fold-change in expression
Days of treatment
n.
DOTLi
Control
Days: UT 1 4 7 1 4 7
cGAS
GAPDH
62 kDa
38 kDa
o.
% cGAS+ MN
MCF10A
HeLa
U2OS
% cGAS+ MN of total
0.0064
0.0073
0.0075
None
DOT1Li
p.
% mCherry-cGAS+ MN
% mCherry-cGAS+ MN of total
0.015
IR
IR+DOT1Li
q.
% cGAS+ MN
MMS
HU
SRSF1 KD
DRB
% cGAS+ MN of total
0.00079
0.0015
0.45
0.26
+DOT1Li
r.
% FLAG-cGAS+ or H3K79me2+ MN
H3K79me2 total
FLAG-cGAS total
% of total MN
0.00045
0.0065
DMSO
DOT1Li
s.
% FLAG-cGAS+ and/or H3K79me2+ MN
FLAG-cGAS only
H3K79me2 only
Both
Neither
% of total MN
0.027
0.12
0.0001
0.0028
DMSO
DOT1Li
t.
STING expression over 7 days of treatment
DOT1Li
Control compound
p = 0.042
p = 0.6
Fold-change in expression
Days of treatment

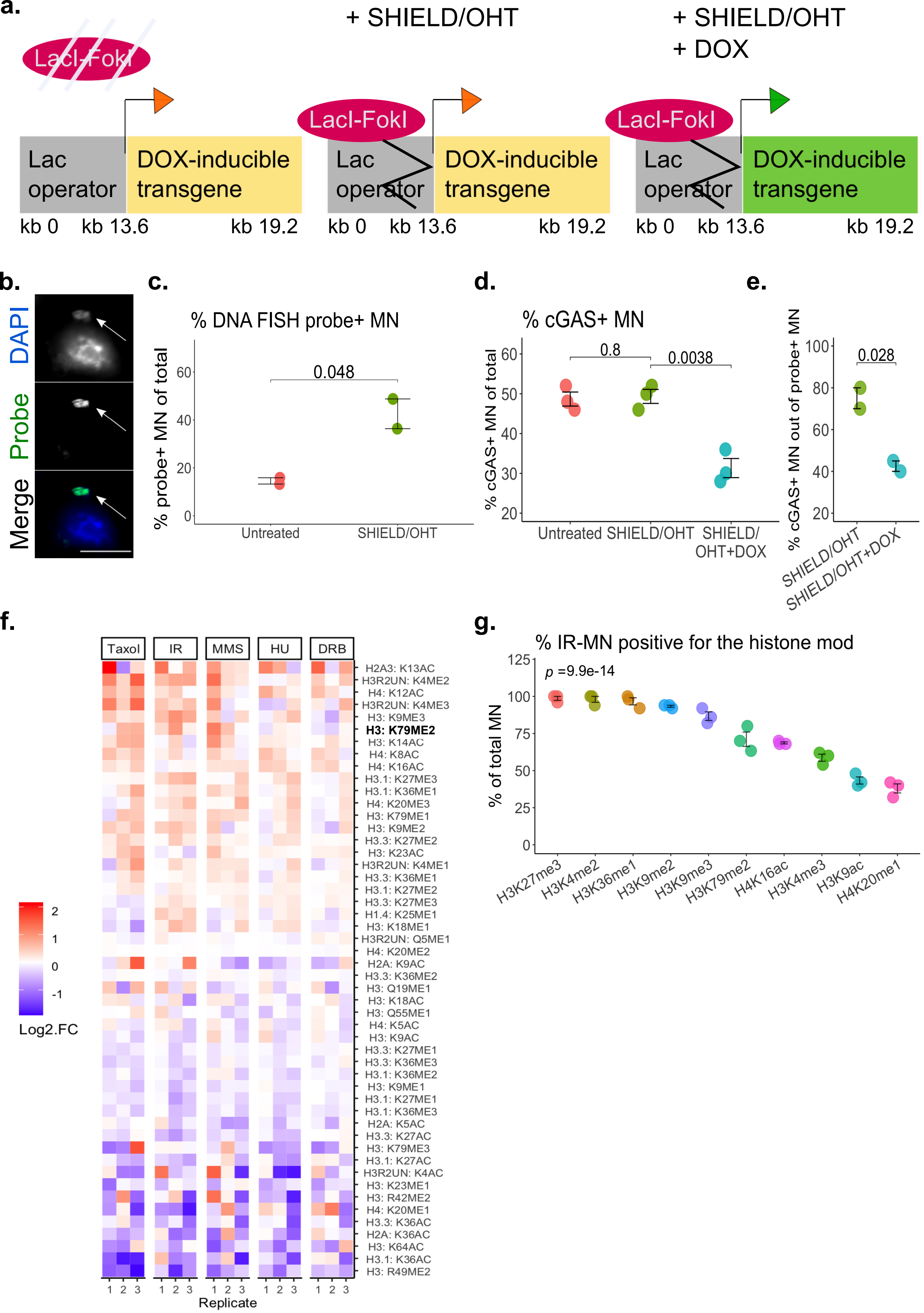
a.
+ SHIELD/OHT
+ SHIELD/OHT
+ DOX
LacI-FokI
Lac operator
DOX-inducible transgene
kb 0
kb 13.6
kb 19.2
b.
DAPI
Probe
Merge
c.
% DNA FISH probe+ MN
% probe+ MN of total
0.048
Untreated
SHIELD/OHT
d.
% cGAS+ MN
% cGAS+ MN of total
0.8
0.0038
Untreated
SHIELD/OHT
SHIELD/OHT+DOX
e.
% cGAS+ MN out of probe+ MN
0.028
SHIELD/OHT
SHIELD/OHT+DOX
f.
Taxol
IR
MMS
HU
DRB
H2A3: K13AC
H3R2UN: K4ME2
H4: K12AC
H3R2UN: K4ME3
H3: K9ME3
H3: K79ME2
H3: K14AC
H4: K8AC
H4: K16AC
H3.1: K27ME3
H3.1: K36ME1
H4: K20ME3
H3: K79ME1
H3: K9ME2
H3.3: K27ME2
H3: K23AC
H3R2UN: K4ME1
H3.3: K36ME1
H3.1: K27ME2
H3.3: K27ME3
H1.4: K25ME1
H3: K18ME1
H3R2UN: Q5ME1
H4: K20ME2
H2A: K9AC
H3.3: K36ME2
H3: Q19ME1
H3: K18AC
H3: Q55ME1
H4: K5AC
H3: K9AC
H3.3: K27ME1
H3.3: K36ME3
H3.1: K36ME2
H3: K9ME1
H3.1: K27ME1
H3.1: K36ME3
H2A: K5AC
H3.3: K27AC
H3: K79ME3
H3.1: K27AC
H3R2UN: K4AC
H3: K23ME1
H3: R42ME2
H4: K20ME1
H3.3: K36AC
H2A: K36AC
H3: K64AC
H3.1: K36AC
H3: R49ME2
Log2.FC
Replicate
g.
% IR-MN positive for the histone mod
p =9.9e-14
% of total MN
H3K27me3
H3K4me2
H3K36me1
H3K9me2
H3K9me3
H3K79me2
H4K16ac
H3K4me3
H3K9ac
H4K20me1

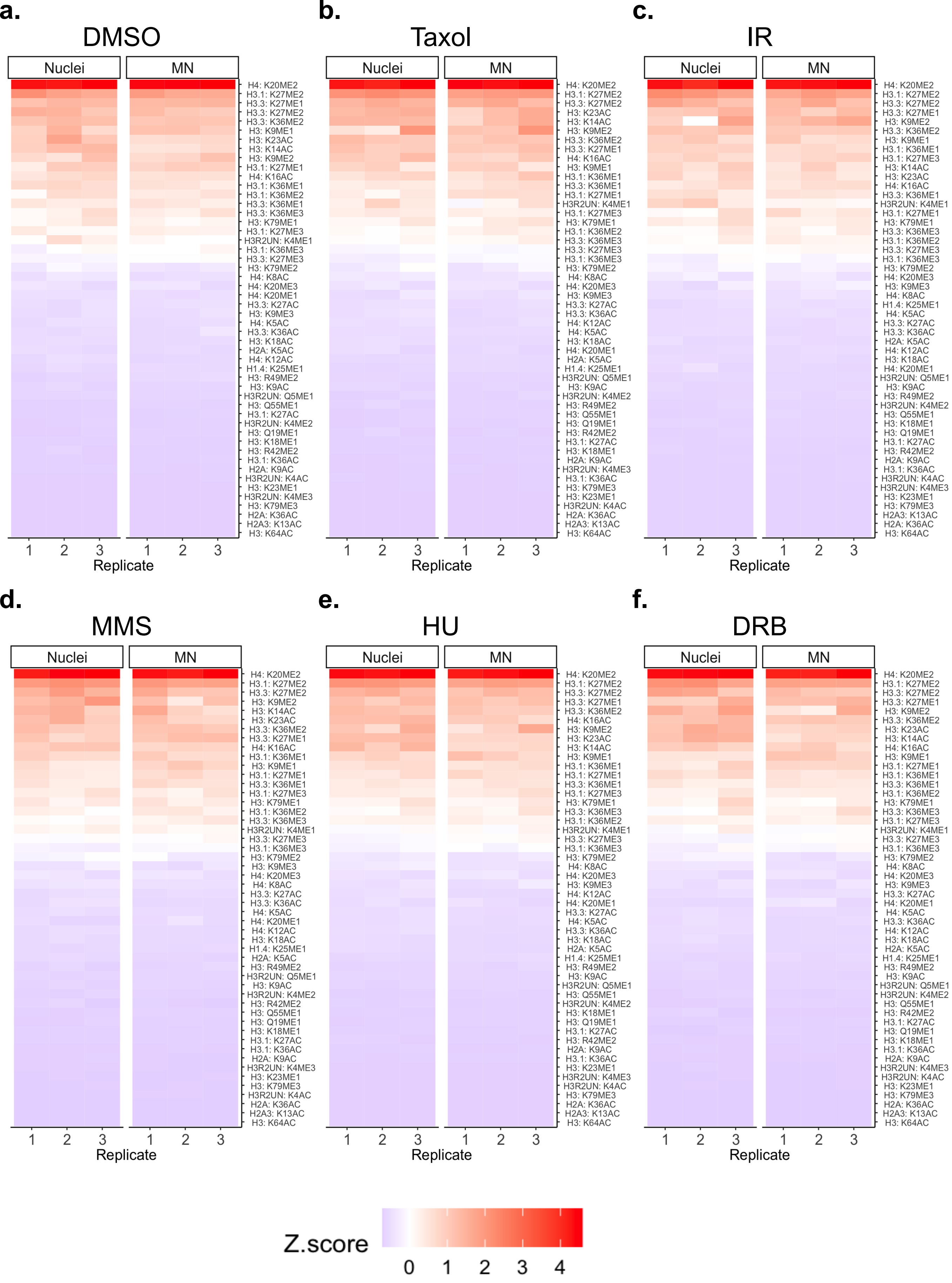
a.
DMSO
Nuclei
MN
H4: K20ME2
H3.1: K27ME2
H3.3: K27ME1
H3.3: K27ME2
H3.3: K36ME2
H3: K9ME1
H3: K23AC
H3: K14AC
H3: K9ME2
H3.1: K27ME1
H4: K16AC
H3.1: K36ME1
H3.1: K36ME2
H3.3: K36ME1
H3.3: K36ME3
H3: K79ME1
H3.1: K27ME3
H3R2UN: K4ME1
H3.1: K36ME3
H3.3: K27ME3
H3: K79ME2
H4: K8AC
H4: K20ME3
H4: K20ME1
H3.3: K27AC
H3: K9ME3
H4: K5AC
H3.3: K36AC
H3: K18AC
H2A: K5AC
H4: K12AC
H1.4: K25ME1
H3: R49ME2
H3: K9AC
H3R2UN: Q5ME1
H3: Q55ME1
H3.1: K27AC
H3R2UN: K4ME2
H3: Q19ME1
H3: K18ME1
H3: R42ME2
H3.1: K36AC
H2A: K9AC
H3R2UN: K4AC
H3: K23ME1
H3R2UN: K4ME3
H3: K79ME3
H2A: K36AC
H2A3: K13AC
H3: K64AC
1 2 3 1 2 3
Replicate
b.
Taxol
Nuclei
MN
H4: K20ME2
H3.1: K27ME2
H3.3: K27ME2
H3: K23AC
H3: K14AC
H3: K9ME2
H3.3: K36ME2
H3.3: K27ME1
H4: K16AC
H3: K9ME1
H3.1: K36ME1
H3.3: K36ME1
H3.1: K27ME1
H3R2UN: K4ME1
H3.1: K27ME3
H3: K79ME1
H3.1: K36ME2
H3.3: K36ME3
H3.3: K27ME3
H3.1: K36ME3
H3: K79ME2
H4: K8AC
H4: K20ME3
H3: K9ME3
H3.3: K27AC
H3.3: K36AC
H4: K12AC
H4: K5AC
H3: K18AC
H4: K20ME1
H2A: K5AC
H1.4: K25ME1
H3R2UN: Q5ME1
H3: K9AC
H3R2UN: K4ME2
H3: R49ME2
H3: Q55ME1
H3: Q19ME1
H3: R42ME2
H3.1: K27AC
H3: K18ME1
H2A: K9AC
H3R2UN: K4ME3
H3.1: K36AC
H3: K79ME3
H3: K23ME1
H3R2UN: K4AC
H2A: K36AC
H2A3: K13AC
H3: K64AC
1 2 3 1 2 3
Replicate
c.
IR
Nuclei
MN
H4: K20ME2
H3.1: K27ME2
H3.3: K27ME2
H3.3: K27ME1
H3: K9ME2
H3.3: K36ME2
H3: K9ME1
H3.1: K36ME1
H3.1: K27ME3
H3: K14AC
H3: K23AC
H4: K16AC
H3.3: K36ME1
H3R2UN: K4ME1
H3.1: K27ME1
H3: K79ME1
H3.3: K36ME3
H3.1: K36ME2
H3.3: K27ME3
H3.1: K36ME3
H3: K79ME2
H4: K20ME3
H3: K9ME3
H4: K8AC
H1.4: K25ME1
H4: K5AC
H3.3: K27AC
H3.3: K36AC
H2A: K5AC
H4: K12AC
H3: K18AC
H4: K20ME1
H3R2UN: Q5ME1
H3: K9AC
H3: R49ME2
H3R2UN: K4ME2
H3: Q55ME1
H3: K18ME1
H3: Q19ME1
H3.1: K27AC
H3: R42ME2
H2A: K9AC
H3.1: K36AC
H3R2UN: K4AC
H3R2UN: K4ME3
H3: K23ME1
H3: K79ME3
H2A3: K13AC
H2A: K36AC
H3: K64AC
1 2 3 1 2 3
Replicate
d.
MMS
Nuclei
MN
H4: K20ME2
H3.1: K27ME2
H3.3: K27ME2
H3: K9ME2
H3: K14AC
H3: K23AC
H3.3: K36ME2
H3.3: K27ME1
H4: K16AC
H3.1: K36ME1
H3: K9ME1
H3.1: K27ME1
H3.3: K36ME1
H3.1: K27ME3
H3: K79ME1
H3.1: K36ME2
H3.3: K36ME3
H3R2UN: K4ME1
H3.3: K27ME3
H3.1: K36ME3
H3: K79ME2
H3: K9ME3
H4: K20ME3
H4: K8AC
H3.3: K27AC
H3.3: K36AC
H4: K5AC
H4: K20ME1
H4: K12AC
H3: K18AC
H1.4: K25ME1
H2A: K5AC
H3: R49ME2
H3R2UN: Q5ME1
H3: K9AC
H3R2UN: K4ME2
H3: R42ME2
H3: Q55ME1
H3: Q19ME1
H3: K18ME1
H3.1: K27AC
H3.1: K36AC
H2A: K9AC
H3R2UN: K4ME3
H3: K23ME1
H3: K79ME3
H3R2UN: K4AC
H2A: K36AC
H2A3: K13AC
H3: K64AC
1 2 3 1 2 3
Replicate
e.
HU
Nuclei
MN
H4: K20ME2
H3.1: K27ME2
H3.3: K27ME2
H3.3: K27ME1
H3.3: K36ME2
H4: K16AC
H3: K9ME2
H3: K23AC
H3: K14AC
H3: K9ME1
H3.1: K36ME1
H3.1: K27ME1
H3.3: K36ME1
H3.1: K27ME3
H3: K79ME1
H3.3: K36ME3
H3.1: K36ME2
H3R2UN: K4ME1
H3.3: K27ME3
H3.1: K36ME3
H3: K79ME2
H4: K8AC
H4: K20ME3
H3: K9ME3
H4: K12AC
H4: K20ME1
H3.3: K27AC
H4: K5AC
H3.3: K36AC
H3: K18AC
H2A: K5AC
H1.4: K25ME1
H3: R49ME2
H3: K9AC
H3R2UN: Q5ME1
H3: Q55ME1
H3R2UN: K4ME2
H3: K18ME1
H3: Q19ME1
H3.1: K27AC
H3: R42ME2
H2A: K9AC
H3.1: K36AC
H3: K23ME1
H3R2UN: K4ME3
H3R2UN: K4AC
H3: K79ME3
H2A: K36AC
H2A3: K13AC
H3: K64AC
1 2 3 1 2 3
Replicate
f.
DRB
Nuclei
MN
H4: K20ME2
H3.1: K27ME2
H3.3: K27ME2
H3.3: K27ME1
H3: K9ME2
H3.3: K36ME2
H3: K23AC
H3: K14AC
H4: K16AC
H3: K9ME1
H3.1: K27ME1
H3.1: K36ME1
H3.3: K36ME1
H3.1: K36ME2
H3: K79ME1
H3.3: K36ME3
H3.1: K27ME3
H3R2UN: K4ME1
H3.3: K27ME3
H3.1: K36ME3
H3: K79ME2
H4: K8AC
H4: K20ME3
H3: K9ME3
H3.3: K27AC
H4: K20ME1
H4: K5AC
H3.3: K36AC
H4: K12AC
H3: K18AC
H2A: K5AC
H1.4: K25ME1
H3: R49ME2
H3: K9AC
H3R2UN: Q5ME1
H3R2UN: K4ME2
H3: Q55ME1
H3: R42ME2
H3.1: K27AC
H3: Q19ME1
H3: K18ME1
H3.1: K36AC
H2A: K9AC
H3R2UN: K4ME3
H3R2UN: K4AC
H3: K23ME1
H3: K79ME3
H2A: K36AC
H2A3: K13AC
H3: K64AC
1 2 3 1 2 3
Replicate
Z.score
0 1 2 3 4

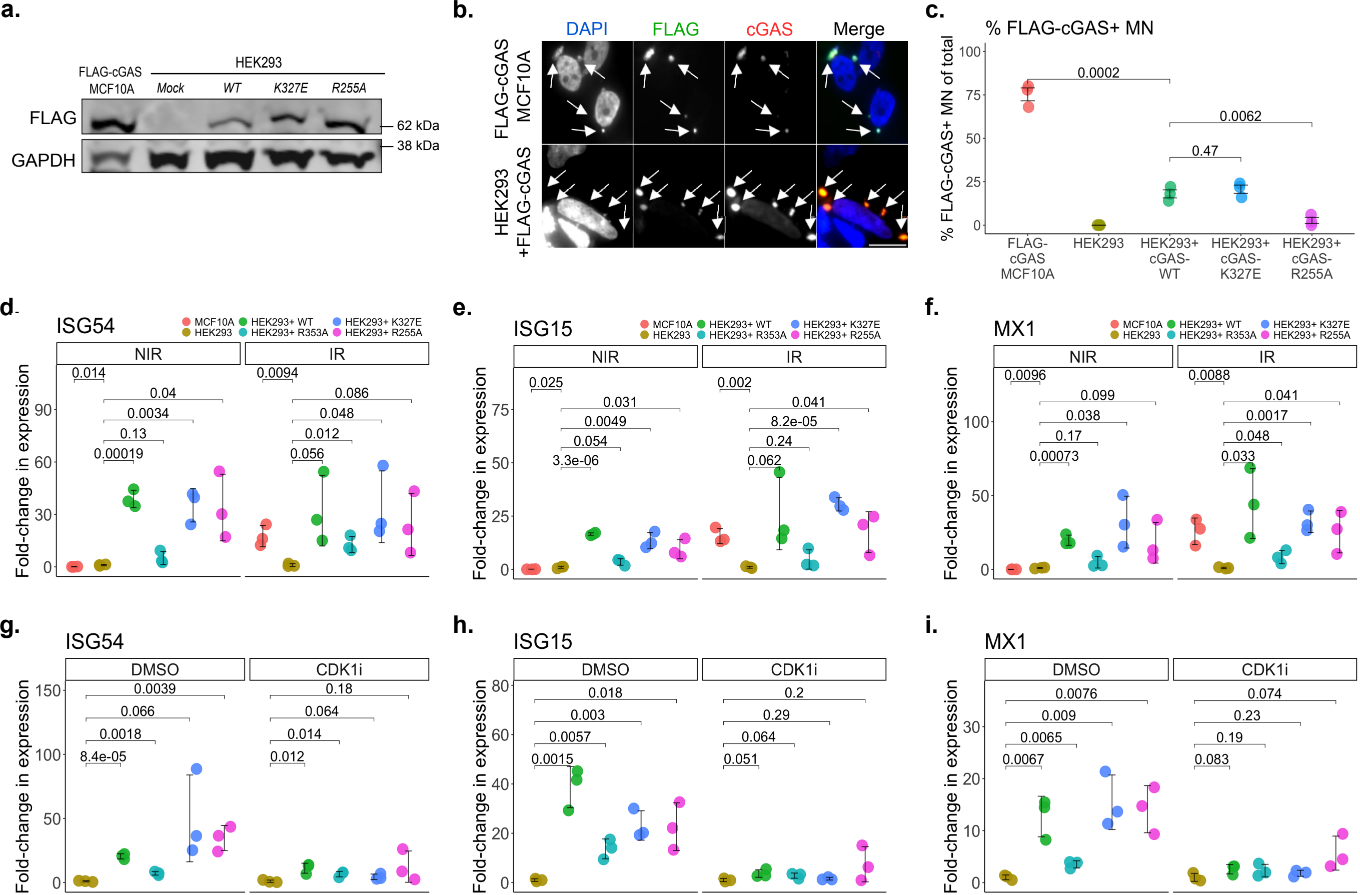
a.
FLAG-cGAS MCF10A
HEK293
Mock
WT
K327E
R255A
FLAG
GAPDH
62 kDa
38 kDa
b.
DAPI
FLAG
cGAS
Merge
FLAG-cGAS MCF10A
HEK293 +FLAG-cGAS
c.
% FLAG-cGAS+ MN
% FLAG-cGAS+ MN of total
0.0002
0.0062
0.47
FLAG-cGAS MCF10A
HEK293
HEK293+ cGAS-WT
HEK293+ cGAS-K327E
HEK293+ cGAS-R255A
d.
ISG54
MCF10A
HEK293
HEK293+ WT
HEK293+ R353A
HEK293+ K327E
HEK293+ R255A
NIR
IR
Fold-change in expression
0.014
0.04
0.0034
0.13
0.00019
0.0094
0.086
0.048
0.012
0.056
e.
ISG15
0.025
0.031
0.0049
0.054
3.3e-06
0.002
0.041
8.2e-05
0.24
0.062
f.
MX1
0.0096
0.099
0.038
0.17
0.00073
0.0088
0.041
0.0017
0.048
0.033
g.
ISG54
DMSO
CDK1i
0.0039
0.066
0.0018
8.4e-05
0.18
0.064
0.014
0.012
h.
ISG15
0.018
0.003
0.0057
0.0015
0.2
0.29
0.064
0.051
i.
MX1
0.0076
0.009
0.0065
0.0067
0.074
0.23
0.19
0.083

Figure S1. Variable recognition of MN across genotoxic stress exposures is not an effect of MN burden or changes to cGAS protein availability. (**a**) Western blot for SRSF1 expression 48 hours after siRNA application. (**b**) Ratio of MN to nuclei in a microscopy field of view (FOV, top), or percent cGAS+ MN by immunofluorescence (IF; bottom), 72 hours following treatment. (**c**) Percent cGAS+ MN by IF, 72 hours post-exposure in MCF10A, HeLa, or U2OS cells. (**d**) RT-qPCR for *cGAS* and (**e**) Western blot for cGAS expression 72 hours post-exposure. (**f**) Hours a micronucleated cell spends in its next full interphase following each of the indicated exposures. mCherry-H2B live-cell imaging. Each point represents the mean number of hours for 50 cells per biological replicate. (**g**) Linear regression comparing the ratio of MN to nuclei in a microscopy FOV to the % cGAS+ MN by IF, 72 hours post-exposure. Vertical error bars represent MN number, horizontal error bars represent cGAS+ MN. (**h**) Untreated MCF10A and HeLa cells in interphase. Scale bar = 20 μm. (**i**) Percentage of mCherry-cGAS+ MN at the time of their formation and prior to their rupture. Live cell imaging of the mitosis following MN formation. (**j**) Time from MN formation until MN rupture, in the next full interphase following treatment. Live cell-imaging in mCherry-H2B, GFP-NLS-expressing cells. Each point represents the mean number of hours for 50 total cells per biological replicate. (**k**) cGAMP abundance 72 hours following the indicated exposure. Statistical comparisons in (b), (d), (j) by one-way ANOVA. All other statistical comparisons by two-sided Student's t-test. ns: $p > 0.05$, *: $p <= 0.05$, **: $p <= 0.01$, ***: $p <= 0.001$, ****: $p <= 0.0001$. All experiments performed on MCF10A cells unless otherwise indicated. All individual data points presented for immunofluorescent scoring of MN represent the mean percentage of cGAS+ MN, from each biological replicate and 50 total MN per replicate. All individual data points presented for the ratio MN:Nuclei represents the mean of at least 5 FOVs per replicate. All error bars represent standard error of the mean, for three independent biological replicates. All blots and micrographs representative of 3-5 independent experiments. Source data are provided as a Source Data file.

Figure S2. Validating the effects of DOT1Li on % cGAS+ MN following ionizing radiation (IR). (**a**) Linear regression comparing the number of MN in a microscopy field of view to the % cGAS+ MN observed by immunofluorescence (IF). Vertical error bars represent MN number, horizontal error bars represent cGAS+ MN. (**b**) Percent cGAS+ MN by IF, 72 hours post-DRB exposure. Statistical comparisons use DMSO-treatment as the reference group. Displaying Bonferroni-adjusted p-values. (**c**) Percent cGAS+ MN by IF. (**d**) H3K79me2 levels following SCG0946 (DOT1L inhibitor) or SGC0649 (control compound). (**e**) H3K79me2 in HeLa cells following SCG0946 (DOT1L inhibitor) or SGC0649 (control compound). (**f**) H3K79me2 levels in DOT1Li-treated cells. (**g**) Percent cGAS+ MN by IF. (**h**) H3K79me2 in cells treated with DOT1Li, small hairpin (sh)RNA for DOT1L, or shLuciferase control (shLuc). (**i**) Percent cGAS+ MN by IF. (**j**) H3K79me2 levels in DOT1Li- or siRNA-treated cells. (**k**) Percent cGAS+ MN by IF. (**l**) Percent ruptured MN by IF. (**m**) RT-qPCR for *cGAS* and (**n**) Western blot for cGAS. Statistical comparison in (m) by one-way ANOVA. (**o**) Percent cGAS+ MN by IF. (**p**) Percent mCherry-cGAS+ MN by IF. (**q**) Percent cGAS+ MN by IF. (**r**) Total percent FLAG-cGAS+ or H3K79me2+ MN by IF. (**s**) Percent single-positive FLAG-cGAS+ MN, single-positive H3K79me2+ MN, double-positive MN, or double-negative MN by IF. (**t**) *STING* expression by RT-qPCR. Statistical comparison by one-way ANOVA. All other statistical comparisons by two-sided Students t-test unless otherwise indicated. ns: $p > 0.05$, *: $p <= 0.05$, **: $p <= 0.01$, ***: $p <= 0.001$, ****: $p <= 0.0001$. All experiments performed on MCF10A cells, 72 hours post-10 Gy IR exposure and following seven-day pre-treatment with SGC0946 unless otherwise indicated. All individual data points presented for immunofluorescent scoring

of MN represent the mean percentage of MN that were positive for the indicated marker, from each biological replicate out of 50 total MN per replicate. All error bars represent standard error of the mean, for three independent biological replicates. All blots and micrographs representative of 2-5 independent experiments. Source data are provided as a Source Data file.

Figure S3. Micronuclei retain chromatin features of the primary nucleus. (a) Schematic of the 256 U2OS reporter system used to create double-stranded breaks at a defined region upstream of a doxycycline (DOX)-inducible transcriptional unit. The addition of SHIELD/OHT directs the FokI nuclease to create a break, and the addition of DOX activates transcription at the nearby locus. **(b)** DNA FISH to verify that SHIELD/OHT-targeted region of the genome is sequestered in resulting MN. SHIELD/OHT-treated 256 U2OS cells, stained with DAPI (blue) and an anti-256 FISH probe followed by FITC-tagged anti-probe secondary (green). Arrow = FISH probe+ MN. Scale bar = 10 μm. Image representative of 3 experiments. **(c)** Percent targeted locus-positive MN by IF, 72 hours post-exposure to SHIELD/OHT in 256 U2OS. **(d-e)** Percent cGAS+ MN by IF in 256 U2OS, 72 hours post-exposure to SHIELD/OHT, with DOX treatment starting twelve hours prior to SHIELD/OHT. Each point represents the mean % cGAS+ MN out of (d) 50 total MN or (e) probe+ MN, per 3 independent biological replicate. **(f)** Heat map displaying histone modifications found by mass spectrometry in HeLa MN six days following treatment with each of the indicated treatment conditions. Log2 fold-change (FC) in abundance of each individual histone modification is calculated relative to the measured abundance of the same histone modification in MN that form following DMSO alone (spontaneously-occurring MN). $N = 3$ independent replicates. **(g)** Percentage of MN positive for the indicated histone modification by immunofluorescence (IF), 72 hours post-exposure to 10 Gy IR. All statistical comparisons by two-sided Students t-test, excepting (G) which used one-way ANOVA. NS: $p = 1$, ns: $p > 0.05$, *: $p <= 0.05$, **: $p <= 0.01$, ***: $p <= 0.001$, ****: $p <= 0$. All individual data points presented for immunofluorescent scoring of MN represent the mean percentage of MN that were positive for the indicated marker, from each biological replicate out of 50 total MN per replicate. All error bars represent standard error of the mean. Source data are provided as a Source Data file.

Figure S4. Micronuclei and nuclei share patterns of histone modifications. (a-f) Histone modifications found by mass spectrometry in HeLa nuclei and MN six days following treatment with each of the indicated treatment conditions. Z-score is calculated separately for each treatment condition. $N = 3$ independent replicates.

Figure S5. The nucleosome-tethering residue of cGAS is necessary for micronuclei localization in HEK293 cells. (a) cGAS expression in HEK293 cells, 72 hours post-transfection with the indicated FLAG-cGAS plasmid. MCF10A cells stably expressing FLAG-cGAS are shown as a control. Image representative of three independent experiments. **(b)** Representative FLAG-cGAS+ MN (arrows) in irradiated MCF10A cells stably expressing a wild-type FLAG-cGAS transgene (top) or non-irradiated HEK293 cells transiently transfected with a wild-type FLAG-cGAS-encoding plasmid (bottom). Images taken five days following plasmid transfection. Scale bar = 20 μm. Images representative of 3 experiments. **(c)** Percent FLAG-cGAS+ MN by IF in HEK293 cells, 72 hrs following 10 Gy IR, transfected with the indicated FLAG-cGAS plasmid 24 hours prior to IR. **(d-f)** Interferon-stimulated gene (*ISG*) expression by RT-qPCR. HEK293 cells were transfected with the indicated FLAG-cGAS-encoding plasmid

five days prior to ISG measurement. 10 Gy IR exposure was applied one day following plasmid transfection. MC10A cells stably expressing wild-type FLAG-cGAS are shown as controls. Statistical comparisons by two-sided Students t-test. ns: $p > 0.05$, *: $p <= 0.05$, **: $p <= 0.01$, ***: $p <= 0.001$, ****: $p <= 0$. All individual data points presented for immunofluorescent scoring of MN represent the mean percentage of MN that were positive for the indicated marker, from each biological replicate out of 50 total MN per replicate. All error bars represent standard error of the mean, for three independent biological replicates. Source data are provided as a Source Data file.

Table S1. Structural Genomics Consortium epigenetic inhibitor library. Information on target protein, inhibitor compound, recommended dose and exposure times for the library used to generate the data presented in Figure 2A and Figure S2B, as set out by the Structural Genomics Consortium (https://www.thesgc.org/chemical-probes).

Compound	Target	Recommended concentration (µM)	Minimum time required for full reduction in in-cell biomarker activity	PMID
GSK8814	ATAD2A/B	1	1h	27530368
BAY-850	ATAD2A	3	1h	29043777
GSK2801	BAZ2A/B	3	1h	25799074
BAZ2-ICR	BAZ2A/B	1	1h	25719566
JQ1	BET	0.2	5h	20871596
BI-9564	BRD9/7	1	1h	26914985
TP-472	BRD9/7	1	18h	26914985
I-BRD9	BRD9	1	18h	25856009
NI-57	BRPF1, BRPF2, BRPF3	1	1h	28714688
PFI-4	BRPF1B	1	1h	26139243
GSK6853	BRPF1B	1	1 hr	27326325
BAY-299	BRPF2(1)/TAF1(2)	1	1 hr	28402630
SGCCBP30	CBP, EP300 bromo	1	1h	24946055
ICBP112	CBP, EP300 bromo	3	1h	26552700
A-485	CBP, EP300 HAT	0.8	3 hr	28953875
NVS-CECR2-1	CECR2	1	1h	28402630
TP-238	CECR2/FALZ	0.3		28402630
GSK4027	GCN5/PCAF	1	18h	28002667
L-Moses	GCN5/PCAF	5	18h	27966810
GSK J4	JMJD3, UTX, JARID1B	5	1d	22842901
GSKLSD1	LSD1	1	1-2d	26175415
GSK484	PAD4	10	3h	26436839
PFI-3	Smarca2 Smarca4, PB1	1	1h	26139243
UNC1215	L3MBTL3	1	3d	23292653
Vinspinin	Spindlin (Tudor like 1 and 2 domain)	0.3		31260300
BI-9321	NSD3 PWWP1	10	1d	31285596
A-395	EED	1	3d	28135237
SGC0946	DOT1L	1	4-14d	23250418
UNC1999	EZH2, EZH1	3	3d	23614352
GSK343	EZH2	3	3d	24900432
UNC0642	G9a, EHMT1	1	3d	24102134
A-366	G9a, EHMT1	1	3d	24900801
MRK-740	PRDM9	3	1d	31848333
MS023	PRMT type I	0.1 - PRMT1; 500 - PRMT6	20 h (PRMT6), 2 days (PRMT1)	26598975
MS049	PRMT4/6	5	20h	31657716
SGC 707	PRMT3	1	1d	25728001
TP-064	PRMT4	1	3d	29712619
SKI-73	PRMT4	1	3d	31657716
GSK591	PRMT5	1	4d	26985292
LLY-283	PRMT5	0.5	q	30034588
SGC3027	PRMT7	3	2d	32409666
PFI-2	SETD7	1	1d	25136132
BAY598	SMYD2	1	1d	27075367
PFI-5	SMYD2	3	1d	31415173
BAY-6035	SMYD3	1	1d	34154424
A-196	SUV420H1/2	1	1-2d	28114273
OICR-9429	WDR5	3	1d	26167872
GSK864	IDH1 mutant	0.3	2d	25622091
